# Supplementary material for: Lay attitudes and misconceptions and their implications for the control of brucellosis in an agro-pastoral community in Kilombero district, Tanzania
Source: PLoS Negl Trop Dis. 2021 Jun 10;15(6):e0009500. doi: 10.1371/journal.pntd.0009500 (PMC8219154; doi:10.1371/journal.pntd.0009500)
Supplement: S1 Structured Interview questionnaire — (DOCX) [file pntd.0009500.s003.docx]

## Appendix 4: Structured Interview Guide

**Household description**

| Region |  |
| --- | --- |
| District |  |
| Ward |  |
| Village |  |
| Sub-village |  |

**Section 1: Demographic Information**

| 1. | Sex | 1. Male 2. Female | CHOOSE ONE |
| --- | --- | --- | --- |
| 2. | Age | 1. 18-25 years 2. 26-33 years 3. 34-41 years 4. 42-49 years 5. >50 years | CHOOSE ONE |
| 3. | Marital Status | 1. Single 2. Married 3. Separated 4. Divorced 5. Widowed | CHOOSE ONE |
| 4. | Highest level of Education | 1. None 2. Primary 3. Secondary 4. College/University 5. Postgraduate | CHOOSE ONE |
| 5. | Religion | 1. Christian 2. Muslim 3. Atheist 4. Traditionalist 5. Other | CHOOSE ONE |
| 6. | Tribe | 1. Wamaasai 2. Wasukuma 3. Wanyakyusa 4. Wasagara 5. Wakuguru 6. Wapogoro 7. Wandamba 8. Wanyamwezi 9. Wamang’ati 10. Warangi 11. Wachagga 12. Other (Specify)…………………………………… | CHOOSE ONE |
| 7. | Main Livelihood Activity | 1. Pastoralism 2. Agro pastoralism 3. Employed 4. Casual Labor 5. Self employed 6. Other (Specify) | CHOOSE ONE |

**Section 2: Knowledge of brucellosis and treatment pathways for human brucellosis**

| ***8.*** | ***Have you ever heard of the disease brucellosis in livestock?***  *If yes go to no 9* | 1. Yes 2. No | CHOOSE ONE |
| --- | --- | --- | --- |
| ***9.*** | ***What are the major signs and symptoms of brucellosis in livestock?*** | 1. Abortions 2. Decreased milk production 3. Birth of weak calves 4. Still births 5. Infertility 6. Weight loss 7. Lameness 8. Swollen joints (hygromas) 9. Others (Please specify) | MARK ALL THAT APPLY |
| 10. | Have you ever heard of the disease brucellosis in humans? *If yes go to No 11* | 1. Yes 2. No | CHOOSE ONE |
|  | **NB:** | **Ask Questions 11 to 20 to only those who answer yes to Q 10** |  |
| 11. | What are the major signs and symptoms of brucellosis in humans? | 1. Fever 2. Chills 3. Headache 4. Joint Pains 5. Abdominal pain 6. General malaise 7. Diarrhea 8. Vomiting 9. Sweating 10. Weight Loss 11. Other (Specify)…………………………. | TICK ALL THAT APPLY |
| 12. | What was the source of this information? | 1. Family 2. Friends 3. Radio 4. TV 5. Posters/Pamphlets 6. School Teacher 7. Health Worker 8. Other (Specify)…………………….. | TICK ALL THAT APPLY |
| 13. | Have you or any of your family members ever suffered from brucellosis? *If yes to No 13, ask the following questions* | 1. Yes 2. No 3. Don’t Know | CHOOSE ONE |
| 14. | If yes what symptoms did, they exhibit? | 1. Fever 2. Chills 3. Headache 4. Joint Pains 5. Diarrhea 6. Abdominal pain 7. Vomiting 8. Sweating 9. Weight Loss 10. *Rash* 11. Other(s)(Specify)…………………………. | TICK ALL THAT APPLY |
| 15. | How do you think you/they contracted brucellosis? | 1. Consuming raw milk 2. Consuming raw blood 3. Consuming raw or poorly cooked meat 4. Direct contact with animal fluids during calving 5. Inhalation of infected aerosols 6. Other (Specify) | TICK ALL THAT APPLY |
| 16. | How did you know it was brucellosis you/they were suffering from? | 1. Self-diagnosis 2. Diagnosis made at a health facility 3. Other(specify) | CHOOSE ONE |
| 17. | What was your occupation or that of the patient at the time when they contracted brucellosis? | 1. Pastoralist 2. Agro pastoralist 3. Housewife 4. Business owner 5. Other (Specify)……………… | CHOOSE ONE |
| 18. | What was the initial course of action you or the patient took when you suspected you had brucellosis? | 1. Visited a health facility 2. Took traditional herbs 3. Visited a healer 4. Home Remedies (Specify)………………. 5. Prayer 6. Over the counter drugs 7. Other (Specify)……………… | CHOOSE ONE |
| 19. | Why did you choose this course? | 1. Most effective 2. Cheapest 3. Nearest 4. Other(specify) | CHOOSE ONE |
| 19. | Were there any subsequent steps you took after that in seeking treatment? | 1. Visited a health facility 2. Took traditional herbs 3. Visited a traditional healer 4. Home remedies (Specify) 5. Prayer 6. Over the counter drugs 7. Other (Specify) 8. *None* | TICK ALL THAT APPLY |
| 20. | In your opinion what is the most effective treatment for human brucellosis? | 1. Traditional herbs 2. Hospital Medicines 3. Home remedies 4. Prayer 5. Other (Please specify) …………… | CHOOSE ONE |
|  | **NB:** | **Ask No 21-23 only to those who responded yes to Q 8 and 10** |  |
| 21. | Can brucellosis be transmitted from wildlife to livestock | 1. Yes 2. No 3. I don’t know | CHOOSE ONE |
|  | If yes how | List…. | LIST THE WAYS |
| 22. | Can brucellosis be transmitted from livestock to humans? ***If yes go to No 23)*** | 1. Yes 2. No 3. I don’t Know | CHOOSE ONE |
| ***23.*** | *If yes, how?* | 1. Consuming raw milk 2. Consuming raw blood 3. Consuming raw or poorly cooked meat 4. Direct contact with animal fluids during calving 5. Inhalation of infected aerosols 6. Other (specify) | TICK ALL THAT APPLY |

**Section 3: Perceived Vulnerability to Brucellosis (**CHOOSE ONE**)**

| **24.** | **Statement on vulnerability to brucellosis** | ***YES*** | ***NO*** | ***SOMETIMES (specify)*** |
| --- | --- | --- | --- | --- |
| a. | We milk our livestock when we know they are sick |  |  |  |
| b. | We consume milk from animals that we know are sick |  |  |  |
| c. | We boil our milk before consumption |  |  |  |
| d. | We slaughter animals that are sick |  |  |  |
| e. | We slaughter dead animals |  |  |  |
| f. | We consume meat from animals that we know were sick |  |  |  |
| g. | We consume meat from dead animals |  |  |  |
| h. | We consume raw blood from animals |  |  |  |
| i. | We consume meat that is raw or has not been cooked properly |  |  |  |
| j. | We reside in the same house with livestock |  |  |  |
| k. | We assist livestock to deliver using our bare hands |  |  |  |
| l. | *We graze our livestock in areas with wild animals* |  |  |  |

| ***25.*** | Has any of your livestock aborted in the last one year? | ***Yes/No (If yes go to No 26)*** |
| --- | --- | --- |
| ***26.*** | Which ones?   1. Cattle 2. Goats 3. Sheep | TICK ALL THAT APPLY |
| ***27.*** | How did you dispose off the aborted material?   1. Fed it to the dogs 2. Buried it 3. Left it to rot on the ground 4. Other (specify) |  |
| ***28.*** | What do you think was the cause(s) of the abortion? | LIST CAUSES |
| ***29.*** | Have you had any cases of retained placenta in your herd in the last one year? | YES/NO (If yes go to Q 30) |
| ***30.*** | Which ones?   1. Cattle 2. Goats 3. Sheep | TICK ALL THAT APPLY |
| ***31.*** | What do you think was the cause(s) of the retained placenta? | LIST CAUSES |
| ***32.*** | Has any of your livestock delivered a still born offspring in the last one year? | YES/NO (If yes go to No 30) |
| ***33.*** | Which ones?   1. Cattle 2. Goats 3. Sheep | TICK ALL THAT APPLY |
| ***34.*** | What do you think caused the still birth? | LIST CAUSES |
| ***35.*** | Have you had any infertile livestock in your herd in the last one year? | YES/NO (If yes go to No 36) |
| ***36.*** | Which ones?   1. Cattle 2. Goats 3. Sheep | TICK ALL THAT APPLY |
| ***37.*** | What do you think was the cause of the infertility? | LIST CAUSES |

***Section 4: Treatment pathways related to febrile illnesses***

| ***38.*** | Have you or any of your family members had a fever in the last three months? | YES/NO (If yes go to No 39***)*** |
| --- | --- | --- |
| ***39.*** | What other symptoms (s) did they exhibit?   1. Fever 2. Chills 3. Headache 4. Joint Pains 5. Diarrhea 6. Abdominal pain 7. Vomiting 8. Sweating 9. Weight Loss 10. Rash 11. Other(s)(Specify)…………………………. | TICK ALL THAT APPLY |
| ***40.*** | What was initial course of action?   1. Visited a health facility 2. Took traditional herbs 3. Visited a healer 4. Home Remedies (Specify)………………. 5. Prayer 6. Over the counter drugs 7. Other (Specify)………………….. | CHOOSE ONE |
| ***41.*** | Were there any subsequent steps you took after that in seeking treatment? | YES/NO (If the answer is yes go to No. 42) |
| ***42.*** | 1. Visited a health facility 2. Took traditional herbs 3. Visited a traditional healer 4. Home remedies (Specify) 5. Prayer 6. Over the counter drugs 7. Other (Specify) | TICK ALL THAT APPLY |

**END**
