# Supplementary material for: Lay attitudes and misconceptions and their implications for the control of brucellosis in an agro-pastoral community in Kilombero district, Tanzania
Source: PLoS Negl Trop Dis. 2021 Jun 10;15(6):e0009500. doi: 10.1371/journal.pntd.0009500 (PMC8219154; doi:10.1371/journal.pntd.0009500)
Supplement: S1 Qualitative tool — (PDF) [file pntd.0009500.s004.pdf]

Appendix 3: Focus group discussion checklist

ID NO:

Demographics:

Type of Group:

No in a Group:

Village:

Theme 1: Animal husbandry

- Describe the livestock practices: (herding, watering, milking, treating, slaughtering, preparation and consumption of animal and animal products, assisting in births, caring for diseased animals, where animals are kept) in terms of **age** and **gender** dynamics. **Probe**: boiling milk, using gloves during assisted parturition, handling aborted foetuses, residing with livestock. **Probe:** List of activities performed per age group

- Describe the livestock movement and grazing patterns. **Probe**: where and in which environments. **Mapping** exercise for spaces: homes, farms, grazing areas, wildlife areas, hospitals, chemists. **Probe**: seasonal variations, wild animals interaction with livestock, which categories of people are involved in this.

Theme 2: Knowledge and Perceptions regarding livestock diseases and brucellosis:

- Describe some of the common livestock diseases in this region and their symptoms. **Free listing** all the diseases of livestock and ranking in order of severity.
- Describe zoonotic diseases. **Probe**: Brucellosis. Risk factors perceptions like consumption of raw milk, handling aborted foetuses, residing with livestock. **Probe:** Age and gender of those most likely to be involved with each risk factor.

- Describe how symptoms like infertility, weak or still born calves, retained placenta, reduced milk yield, hygromas are understood and addressed eg **do they signal a big problem**. **Probe**: Perceptions, treatment strategies.

Theme 3: Treatment pathways for febrile illnesses

- Kindly name the common human diseases in this locality that cause fever. **Free listing** all human diseases and **ranking** in order of severity: **Probe**: Zoonotic diseases. **Probe**: Local names for fever and meaning, undulant fever, other common symptoms, causes, progression, degrees of severity

- What kinds of treatment are sought for febrile illnesses? **Probe**: Who determines treatment sought and that decision making process, Progression in seeking care and duration taken between each method, Differences between males and females, Children and adults, Utilization of health care facilities, Over the counter drugs, testing, alternative treatment sources

- Please tell me about the challenges you encounter in accessing treatment for febrile illnesses. **Probe**: Duration of illness, Cost, Accessibility of treatment, Preferred treatment, gender differences, other work commitments. **Probe**: perceptions related to the health care system.

- Kindly tell me about the difficulties you face after the treatment for a febrile illness. **Probe**: Any recurring symptoms, Actions taken.
