# Supplementary material for: Lay attitudes and misconceptions and their implications for the control of brucellosis in an agro-pastoral community in Kilombero district, Tanzania
Source: PLoS Negl Trop Dis. 2021 Jun 10;15(6):e0009500. doi: 10.1371/journal.pntd.0009500 (PMC8219154; doi:10.1371/journal.pntd.0009500)
Supplement: S1 Qualitative data — (PDF) [file pntd.0009500.s005.pdf]

FGD WOMEN in Siginali on 12/8/2019

Q1 Gender roles in livestock management

Interviewer: What are the duties of men and women that are related to livestock?

Respondent: Men: Herding, Milking, treating, helping cattle to deliver, slaughtering and skinning.

Women: Milking if men busy or few men, herding among the maasai, milking is for women among the maasai, women among the Sukuma treat cattle only if there is no male present at the time and they do it together because its not easy for a woman. For the Sukuma if there is no male present and a cow needs to deliver we call a male neighbor to help us. For us the maasai if the cow is not hostile we assist it ourselves. But if the cow is hostile we look for a man to help. For us women our biggest duties are milking alongside cooking, looking after children, fetching water and looking for firewood.

Interviewer: What about farming?

Respondent: This one we do together both men and women.

Interviewer: Do women also plough using oxen?

Respondent: No this one men do. And sometimes we women do it too. The woman drives the plough while the man drives the oxen. If one is tired, we exchange. The duties are both equal none is harder than the other.

Interviewer: At what age do the boys go to herd?

Respondent: They start from age 7.

Respondent: Oh, no they start much younger from the age of 5 they start looking after calves.

Interviewer: Then when do they start looking after cattle?

Respondent: This they do from age 10 onwards. But if the cattle are many then they do the herding together. One boy cannot manage a lot of cattle on his own.

Interviewer: What age do they go to herd and live alone in the camps?

Respondent: From 10 onwards they can go but often not alone they have someone else older than them. Often between 10-30 years old. Sometimes if there is no male children then the girls will herd too but not to live in the camps.

Interviewer: Ehe.

Respondent: Another problem we have here is our chicken getting sick and dying. This is so hard because chicken belong to us women and are a source of income for us. The chicken show symptoms of whitish diarrhea and swelling in the mouth and eyes. We have tried treating it to no avail. It is commonest in the flooding season and all the chicken often die. It is contagious and spreads very fast.

Interviewer: Have you informed the livestock officers:

Respondent: (Laughter). We do not because as a woman I cannot go by myself and usually the men are only concerned with cattle and shoats which belong to them and for those they go to the livestock officers if they have any challenges. As a woman I cannot go. For men chicken are just birds and if they find one is dying the men say "ah, its okay now we can eat chicken today". But for me as a woman it is very painful because its from selling the chicken that I get some money for buying things like bowls and pots. I could also buy a dress. It's a big loss to me. Men are not concerned about chicken, that is purely a woman's business how to feed them and care for them. If a woman has 10 cocks and you sell them, you get good income. There are people who come here to buy chicken.

Q2 Practices on assisting with parturition

Interviewer: Do you use gloves when you are helping cows to deliver?

Respondent: No we assist them using our bare hands. We do not use any gloves.

Interviewer: Do you think there is any harm in that?

Respondent: To us there is no harm. We just wash our hands sometimes with soap other times without soap.

Respondent: Sometimes if you are out herding (maasai woman) and you assist an animal to deliver sometimes there is no water to wash your hands so you do not wash all day. Or when you take the cattle to water you rinse your hands but still carry the calf or kid home. So sometimes we use soap to wash hands and other times we do not only plain water.

Interviewer: Do you think there is any harm in being close to livestock like when you assist livestock to deliver?

Respondent: No we have never seen anyone affected by that.

Q3 Practices on residing with livestock

Interviewer: Do you reside with livestock?

Respondent: Yes, when they are young. This we do because the cows can trample on the calves. It is not safe for them. This is after two weeks. Also kids and lambs.

Interviewer: Do you think there is any harm to humans through this?

Respondent: None that we have ever witnessed.

Q4 Practices on raw milk consumption, boiling milk and community engagement

Interviewer: Do you boil your milk before consumption?

Respondent: (Pause). We do not boil milk.

Interviewer: Why do you not boil milk?

Respondent: Its just a habit.

Respondent: From childhood we just are not used to boiling milk. We grew up taking raw milk.

Respondent: We just milk our cows, sieve the milk and then drink it (laughter). Even these children are growing up like that.

Interviewer: Have you ever heard of the importance of boiling milk?

Respondent: (Together). Yes, we have.

Interviewer: Where did you get this information?

Respondent: We hear in the health facility at Signal. They tell us to boil milk for our babies. Also in the hotels we see them boiling milk.

Respondent: But for us we prefer to give freshly milked milk to babies and children. To us we think that is the best milk and nutritious too.

(Laughter)

Respondent: You see as soon as you milk you give the baby while the milk is still warm. We prefer that one.

Interviewer: Why do you not like boiled milk?

Respondent: To us that milk has no taste at all.

Respondent: It tastes terrible.

Respondent: That milk is not good, it can even cause diarrhea.

Respondent: Some children also do not like that milk. They know when it has been boiled. Once you boil milk the flavor changes completely. It even has a funny smell once it has been boiled.

Interviewer: How do you perceive the education you receive about boiling milk at the health facility?

Respondent: They say that it is good to boil the milk before feeding the baby to avoid diseases like stomachache and other diseases. However, our children like raw milk and they are used to it.

Respondent: And also once I give my child raw milk they do not suffer from any diarrhea so I still give it to them (laughter). So I continue with the raw milk.

Respondent: The main reason is that we do not see any adverse effects that our children get from taking raw milk. None at all.

Interviewer: So you are saying that at the health facility they tell you that if you give your baby raw milk the baby will have diarrhea. But when you give it the raw milk the child doesn't have diarrhea?

Respondent: Yes, sure (long laughter). So we do not understand.

Respondent: They say also that the child can get TB and cough a lot. But this is not something that we experience. We so not see our children suffering from any of this as a result of raw milk consumption. (laughter).

Respondent: When I take my child to hospital when they are ill they only will be diagnosed with "homa ya kawaida" (regular illness) never TB(laughter). I don't see any TB its always malaria never diarrhea and TB.

Interviewer: Have you ever sought clarification from the hospital management about this?

Respondent: No, I have never done that because I am afraid. I just agree with the nurse that I will boil the milk but she is not going to follow me to my house so once I get home I give the raw milk to my child. (laughter). I don't see any adverse effects and so I cannot change.

Interviewer: Do children have diarrhea in this locality?

Respondent: Yes, they do but it is not because of milk its because they are sick.

Interviewer: How can you tell that is the case?

Respondent: (Laughter). We just know it is illness.

Respondent: Also there are periods when there is no milk at home and so the child isn't consuming any milk but they still suffer from diarrhea. So it cannot be milk.

Respondent: Also what I do I will first give raw milk to my child. If the child has diarrhea, then I will boil milk for him or her but if not I will not.

Respondent: You actually start with boiled milk for a baby and then if the baby has diarrhea you change to raw milk and the child is okay. You start this even from day one or one week. Some babies do not get satisfied with breast milk alone so you give them cow milk very early. You start by boiling because it becomes like mother's milk because boiling kills any harmful organisms in the milk but if the baby resists it through diarrhea then you change to raw milk.

Respondent: And the child on raw milk becomes very big, they add weight fast and are healthy. So it is good for the baby. And this milk has no harm for adults.

Interviewer: Are there any other categories of people who have their milk boiled before consumption?

Respondent: None, only the babies.

Respondent: We the Maasai we do not boil milk even for the little babies. We just wait until the baby is two months old and then we get the baby his or her own milk traditional bottle. We put some milk and add some water to dilute it and feed the baby. We make it light like breast milk. We don't give the baby whole cows milk. We are told to boil milk in the hospital but we don't. We think that the raw milk is best (laughter). Its is healthier and more nutritious.

Interviewer: What kinds of foods do the herds boys eat in the camps?

Respondent: Usually the take raw milk with rice and ugali. They don't boil milk. And fish too but very often milk.

Interviewer: Do they get afflicted in any way as a result of raw milk consumption?

Respondent: No they do not. Actually here at home even those who don't take milk get sick often while those boys do not get sick at all.

Respondent: So we do not believe it when we hear that raw milk causes diseases because we are taking milk all the time and we are well. And our children do not suffer from TB. They get chest infections but these are not as a result of raw milk consumption.

Respondent: Because sometimes for us the Maasai if a child has a severe chest problem we boil milk and give it to the child while it is hot and the chest issue subsides. That milk "burns" the chest and cures the chest but I give the child while it is very hot not when it is cold.

Interviewer: Do you think you can start to boil milk?

Respondent: Please tell us any dangers that arise from raw milk consumption first. So that we also can understand what exactly is the problem with raw milk. Because they tell us all the time but we do not understand. So please explain to us.

Respondent: You know livestock keepers are very argumentative they like to understand and not just to be told. Like they tell us that we should not wean our babies before six months but we do even from the first month especially if the mother does not have enough milk.

Interviewer: Raw milk can cause diseases like brucellosis and TB. Do you think if you were educated better that you would boil milk?

Respondent: Yes, we would because it would be benefiting us.

Interviewer: Which forum would be best for this kind of education?

Respondent: At the clinic because most women take their kids to the clinic. This is the best place and more clarity on what kinds of diseases. Like today you have told us more diseases. So we need more in depth information.

Q5 Practices on herding and attitudes of herdsmen

Interviewer: What month do the cattle go to the camps?

Respondent: Usually from May, June all the way until April of the following year during the heavy rains season.

Q6 Livestock wildlife interface

Interviewer: Are there any wild animals close to the grazing areas?

Respondent: Yes, they are there but they are far. They don't graze in the same area with the cattle. They do not meet.

Respondent: Unless the wildlife come close to the grazing areas then that is the only time they come into close contact.

Interviewer: What wild animals come close to the grazing areas?

Respondent: Lions, elephants, buffaloes, ("sheshe") puku.

Q7 Common livestock diseases and practices related to abortions in cattle

Interviewer: Do you know any livestock diseases?

Respondent: (PAUSE)…..(Sukuma women silent)

Respondent: (Laughter)…Maasai woman…can I really tell you livestock diseases in Swahili…I know the diseases in Maasai. One of them is CBPP, FMD and one where they get bitten by insects. And also in sheep between Sep-Nov they get a short illness with diarrhea and die soon after. To us we say that the hot sun "burns" sheep. For FMD we take the cow out into the sun and leave it there all day tied to a tree and the cow gets better after being in the hot sun all day. But CBPP and the one for flies we use injectable medicines.

Interviewer: What are the signs of CBPP?

Respondent: (Pause). Usually it is coughing.

Respondent: Also rough hair coat and emaciation.

Interviewer: Have you ever heard of any disease that is transmitted from livestock to humans?

Respondent: We have heard of RVF. We heard that if you eat infected meat then you also get RVF but we have never witnessed it.

Interviewer: Have you ever heard of brucellosis:

Respondent: No we have never heard of that one.

Interviewer: How do you dispose off aborted fetus?

Respondent: We feed it to the dogs.

Interviewer: Is the fetus first cooked?

Respondent: No. We just cut it and feed to the dogs. But if the cow aborts at night you will find that the dogs already ate the fetus.

Interviewer: Why do you need to cut the fetus then?

Respondent: Because you need to distribute the fetus to all the dogs.

Respondent: Others say that it is not right to feed a whole fetus to the dogs. Its important to cut it first. I don't know why but they just say it is not right. Usually it is the herds boys who do the cutting but if they are absent then a woman will cut it.

Q8 Perceptions on infertility in cattle

Interviewer: What causes infertility in livestock?

Respondent: We just know it is infertile. That’s it.

Interviewer: What do you do to an infertile cow in your herd?

Respondent: You just keep the cow until it dies. We don’t sell it. You don’t sell an infertile cow. We don’t know why but…

Respondent: But its because God gave me an infertile cow so I should accept that and because I have other cows that are giving birth I should just let the infertile one stay in the herd until it dies. It’s a curse if I sell it because even the healthier ones can die too. The ones I can sell are the ones which have given birth once or twice then they stopped. The one I cannot sell is the “mgumba” but “tasa” I will sell.

Q9 Perceptions on reduced milk production

Interviewer: What causes reduced milk in a cow?

Respondent: The cow has become old that is why. So you don’t milk the cow once it gives birth it just suckles the young. As long as it is suckling I don’t sell the cow it just gives birth and I don’t milk it.

Interviewer: Why do you keep a cow that is not producing milk for you?

Respondent: Because it is reproducing. When it stops I sell it and buy another one.

Q10 Perceptions on hygromas

I: Have you ever seen a cow with swollen joints?

R: Yes, we do and its just a disease to which we buy medicine and treat it.

Q11 Perceptions on retained placenta

Interviewer: What causes retained placenta?

Respondent: Am not sure. You just give salt to the cow to drink diluted with water and after that it comes out.

Q12 Perceptions on weak or still born calf

Interviewer: Why does a cow give birth to a weak or still born calf?

Respondent: (Pause). Its because it was sick. And also God had planned for that too.

Q13 Common human diseases in this locality

Interviewer: What human diseases are a big challenge here?

Respondent: Malaria. Everyone you take to hospital they say they are suffering from malaria. It is the biggest problem here. It’s a big problem. If one is coughing and you take them to hospital they say it is malaria.

Respondent: Also stomachache.

Respondent: Typhoid.

Q14 Signs of febrile illness

Interviewer: What are the signs of a febrile illness?

Respondent: Its starts with feeling cold all over the body and shivering. Then a fever comes and you take them to hospital and after tests they tell you your child is suffering from malaria. You are given three kinds of medicine for the child. Also headache and stomachache.

Respondent: In children it is mainly vomiting although some adults vomit too. Another disease is UTI too.

Q15 Treatment pathways related to febrile illness

Interviewer: What is the first thing you do when you are suffering from a febrile illness?

Respondent: (Maasai woman) If it is me or my child there is a plant ("ng'ochangoko" in Kisukuma and "okaboya" in kimaasai) that I get form the bush and I prepare it. It causes vomiting and we believe that once you vomit then the malaria is gone. Actually if you tell a child that you are going to get this medicine when they are ill they beg you and tell you that they are not ill anymore (laughter). They do not like it at all. They clean you completely after all the vomiting. You vomit for like 15 minutes and feel a lot better if it is not a bad "homa". This one really helps as first aid. However, if I don't or the child does not feel better then we go to the hospital. Sometimes we get better without going to the hospital. The herbal medicine causes you to vomit until some yellowish stuff comes out. Also sometimes we take salty water and that helps too. All this takes away "homa".

Respondent: We also do the same. And vomiting helps to clear away all the malaria. We do this from age four because at that age the child can accept to drink it. The plant smells and tastes horrible and causes vomiting immediately. Young children we take to the hospital immediately after giving them Panadol. And sometimes we get better without going to the hospital.

Interviewer: What kinds of treatment do you receive in the hospital?

Respondent: They do tests and then give you the necessary medication.

Interviewer: Do you buy over the counter drugs?

Respondent: No we do not.

Interviewer: What are the kinds of diseases do they diagnose after testing?

Respondent: If one has a stomachache then they tell you you are suffering from typhoid. And you buy medication. Or also UTI for stomachache. If you are feeling cold they give you malaria drugs "mseto".

Interviewer: Do you buy malaria drugs before seeing a clinician?

Respondent: No we do not. Sometimes we get the treatment and after using it for a while we still are not feeling better so we go back they test again and tell you you have typhoid and change the drugs. Typhoid they say the symptoms are fatigue and weariness.

Interviewer: Do you go to Kibaoni or St Francis hospitals?

Respondent: Yes, but as a referral from here in Siginali. We do not go directly from here. In Kibaoni we get additional tests.

Interviewer: What are the challenges you face in seeking healthcare?

Respondent: Our biggest challenge is lack of money because you cannot be treated without funds. The only ones who are treated for free are the pregnant women and the children below five years of age. And they also ask you why you do not have insurance. Plus, we also have to buy drugs and they recommend you to go buy drugs in a specific pharmacy.

Respondent: The queues also can be very long in the hospital too and you stay there all day. During the rainy season also we have a big challenge because the roads are impassable. So you keep falling and its really tough. Even motorbikes cannot go across. Pregnant women also have a big challenge in March- April because of mobility especially when one is in labor. When it rains this place floods and there is no clear road and so no mode of transport is usable. If you have a sick person or a mother in labor its really hard. Many women deliver before they get to the hospital. But when its not flooded its okay because the motorbikes come all the way here.

FGD Women Sagamaganga

18/8/2019

Q1 Gender roles in livestock management

Interviewer: Why do you keep livestock?

Respondent: (Pause). We like keeping livestock because they help us to meet our needs. We like to keep sheep, goats, chicken, cattle because goats and sheep help us in lesser problems. You sell and get cash and cattle for bigger challenges like if a family member is hospitalized.

Interviewer: Who is mainly involved in livestock activities like herding?

Respondent: Mainly men but women herd too like goats once in a while. Men also milk though women do that too. Men treat the animals although we assist the men sometimes and as women we can also assist one another to treat the cattle. Some of us know how to even inject cows and calves. We do this though when the men are unavailable. Men slaughter and skin too. Women don't, but in some cases women slaughter and skin too. We assist with parturition. Men do it often but we assist if need be.

Interviewer: At what age do boys start going to herd?

Respondent: Usually it depends. Boys start with looking after calves from age 9. And from then on until 12 they can even look after cattle as long as they are not many. Some people have up to 200 heads of cattle and a boy of 12 can't manage to look after all of them. Boys of 5 years of age look after kids and lambs.

Respondent: The young boys just go along with the older boys to play like the five year olds. Its just getting familiar with herding. So a five-year-old will follow a nine-year-old along to the pasture.

Interviewer: At what age do they start going to the grazing area?

Respondent: From 12 years of age they are able to go. But usually they will not be alone and will be assisted by any of the older boys or men since the herdsmen camps are not far form each other.

Interviewer: So you are saying that women are mainly involved in milking activities?

Respondent: Yes, usually they assist with milking largely.

Interviewer: Do older men also herd?

Respondent: They do if there are no younger men to do it but if they do have younger people they don’t.

Respondent: (Over 60-year-old woman) We do herd like I herd the calves and my husband the cattle if we have no help at home(laughter). If there are no herdsmen, then we have to do it. You meet at home later both husband and wife and you’re so tired. Then in the evening you come home, the wife goes to the kitchen to cook while the husband goes out to milk (more laughter). When you both are so tired then the husband tells the wife that it is time to seek hired help. Or you ask for someone to lend you their child to help you.

Interviewer: What are the main duties of women therefore in general?

Respondent: Milking, cooking, farm work, child care and other household chores.

Q2 Practices on assisting with parturition

Interviewer: Do you use gloves during assisted parturition?

Respondent: No we don't. We do it with our bare hands. If a cow needs help at midnight and the cattle are in the grazing area where do you go to buy the gloves at that time? They just use bare hands.

Interviewer: Do you think there is any danger in that?

Respondent: No, there isn't as long as after you are done you wash your hands.

Respondent: If you don't wash your hands there will be harm because you have touched "dirty fluids" and if you go cook or eat without washing hands you will feel uncomfortable.

Respondent: Yes, its dangerous because if you have a wound on your hand the birth fluids can get into your body and cause illness. However, we have never witnessed any dangers.

Interviewer: Do the herds boys also wash their hands, do they have soap?

Respondent: Yes, they know they should wash their hands and yes they carry soap.

Interviewer: Are gloves available?

Respondent: Yes, they are available in drugs shops but we are not use to wearing them.

Respondent: Indeed, because we grew up seeing our fathers and mothers not use gloves so we also don't use them. And we have never seen any harm arising from this practice.

Q3 Practices on residing with livestock

Interviewer: Do people sleep with or close to livestock?

Respondent: No it doesn't happen. We build shelters for goats and sheep away form the dwellings of people.

Q4 Perceptions on raw milk consumption, boiling milk and community engagement

Interviewer: I know you don't boil milk before consumption, why don't you do it?

Respondent: Some say milk looses flavor when it is boiled (laughter).

Respondent: Boiled milk has a bad taste and smell so all we do is sieve off the dirt and drink the milk raw and fresh or ferment it and consume.

Interviewer: So the biggest challenge Is the bad smell and taste?

Respondent: Yes, that the main reason. We only sieve it to remove the hairs and dirt.

Respondent: However, some people like boiled milk and they do. Some say they have stomach ulcers and boiled milk helps. This is advice they receive from hospital. We don't boil all the milk only for those who like boiled milk or have stomach ulcers. And we love milk.

Respondent: You can also boil milk first before fermenting it but some people do not like it because of the taste. I do not like boiled milk. It has a bad taste and smell.

Interviewer: Do you think there is any danger to peoples health when they consume raw milk?

Respondent: We have never experienced that.

Respondent: Yes, there is danger. There was a time they were saying that there is a diseases arising from raw milk consumption called rift valley fever and so during that time we were boiling milk. So after that time some people got used to consuming boiled milk but some of us have not adapted to that yet. I don't take boiled milk.

Respondent: For some they did not boil milk even during that period of RVF. Only a few people did.

Respondent: I like boiled milk from that time and I don't take raw milk anymore.

Respondent: There are dangers. I have heard that people can get TB from consuming raw milk. That is what I hear. That fresh milk needs to be boiled before consumption and that there are germs in milk that only die when milk is boiled. I hear about that.

Respondent: I have never heard of any dangers.

Interview: What do you think is the reason people don't boil milk even those who have heard that there is potential for human illnesses occasioned by raw milk consumption?

Respondent: Its just that it is not in our custom to boil milk.

Respondent: We also take it lightly that milk would have any harmful effect.

Respondent: Plus, we don't get sick so we wonder how then can it be harmful. I've consumed raw milk since I was a child and I have never experienced any negative effects. Once we see the effects of raw milk consumption and get sick from it then we will start to boil (laughter). But as it is we have not experienced any one person who has become sick from raw milk consumption.

Respondent: Even RVF we never saw any cases here at all but in other regions they did as we heard.

Respondent: (Laughter). They tell us but some of us can't stand boiled milk especially if it is fermented. Also when you boil milk the milk doesn't ferment well and so you cannot obtain the butter ("samuli". Although some people prefer boiled milk and they are used to it and we boil for them. Little babies below four months are given boiled milk but after that age they refuse to take the boiled milk and once you give them the raw milk they drink it. Boiled milk is better for babies because their tummies are weak and cant handle the raw milk and in the boiled milk the germs have been killed.

Interviewer: Do clinicians advise you to boil milk in the health facilities?

Respondent: Yes, they do. They tell us to boil milk. I have not been told that myself but others have.

Respondent: (Laughter). If I fell sick and then I was told in the hospital that raw milk had made me sick, then I would start to boil. But if I have never fallen ill as a result of raw milk consumption then I will continue taking the raw milk. We also find boiling milk to be unnecessary trouble and extra work. So for me I am alone at home and I have a lot to do how then do I start boiling milk? Its too much trouble. So all I do is sieve the milk and put in a clean container for it to ferment and then I consume it. Boiling milk is too much work because the raw milk is not harmful in the first place. However, if I realized that we are getting sick from raw milk consumption and my child is getting sick then I will train myself to boil the milk. But those illnesses are not there, if we see them then we will change our habits too.

Respondent: Yes, we hear about the need to boil milk from both clinicians in the health facilities and the radio. However, we don't take it seriously and we don't really believe that raw milk is harmful. We are used to consuming raw milk. How do we change our behavior as adults now? Sometimes we try but we can't stand it. The fresh milk we can boil but we can't stand boiled milk that has been fermented.

Interviewer: What do you think is the best way to teach people here on the importance of boiling milk?

Respondent: Let me first ask you since you are an expert, does raw milk cause any harm? Which diseases?

Interviewer: Yes, consuming raw milk can have adverse effects to people's health and cause brucellosis, RVF and TB because there are diseases that can be passed from livestock to humans.

Interviewer: What are some of the ways we can educate local people about boiling milk?

(Pause)….

Respondent: To help people understand even if they don't change their habits…people don't get educated through the radio. The best way is to have workshops like in the health facility and educate people. Those who want to be educated will.

Respondent: Its also important to teach with examples meaning that you explain in more in depth detail. This information can also be given to the village heads and they can give it during village meetings as well as in the health facilities as she said.

Q5 Practices on herding and attitudes of herdsmen

Interviewer: When do herds boys take cattle to the grazing area and camps?

Respondent: From June all the way to when the are floods from Feb-March.

Q6 Livestock wildlife interaction

Interviewer: Are there any wild animals in the grazing area?

Respondent: Yes, but far off in the Selous game reserve. But sometimes they come to where cattle are e.g. hyenas. Buffaloes none but “sheshe” do when it floods because they also relocate to higher ground and you can see them in the areas around like in the farms and they graze in the same areas with cattle. During that time, we see them around a lot.

Interviewer: Do you think there is any danger in wild animals and livestock interacting:

Respondent: No, there is no danger at all. The only animal we are wary off is the hyenas because they prey on cattle. But the “sheshe” …in English puku do not cause any harm. These are as big as goats with long curved horns and they graze freely with our cattle.

Q7 Common livestock diseases and practices related to abortions in cattle

Interviewer: What are the common livestock diseases in this area?

Respondent: CBPP and the signs are coughing.

(Pause)

Respondent: There is CBPP and another one…(she consults in Kisukuma)….there is one where there are sores on the coat of the animal. Those are the two main diseases. And also when a cow appears “mad” and gets weak. I don’t know the name is Swahili but in Kisukuma we call it “ndendera”. There is also “lusoona” FMD. FMD the cattle have sores on the hooves and mouth.

(Pause)

Interviewer: Are there treatments for these diseases? R: Yes, they treat them.

(pause). Once they notice the symptoms they treat them quickly because if they don’t the cattle die.

Interviewer: Have you ever heard of the disease brucellosis?

Respondent: (Pause) We have never heard. We know the disease where cattle suffer abortions but we don’t know brucellosis…we call it (consultation in Kisukuma)……we say that the cow has been bitten by tse tse flies. They look like flies but they are bigger in size. When they bite cattle then they suffer abortions and also the cow produces no milk too.

Respondent: Yes, they become weak even if they are able to give birth, they also give birth to a weak calf and have reduced milk production.

Interviewer: So you have heard of the disease that causes abortions but you have never heard of brucellosis?

Respondent: Yes.

Interviewer: Are these diseases seasonal? Or all the time?

Respondent: Usually during the start of the heavy rains that is when the tse tse flies are many.

Respondent: FMD is commonest during the flooding season.

Interviewer: Do you think its harmful when people come into contact with the fluids when a cow aborts or gives birth?

Respondent: We don't come into contact with an aborted fetus. A cow just aborts maybe at 3 or 4 months and you just notice after its over. You don't need to handle it.

Interviewer: What happens to the aborted fetus if its seven months or thereabouts?

Respondent: Its just thrown to the dogs and its cut into pieces first. Usually it's the boys who herd who cut it into pieces like the 10- 12 year olds.

Respondent: Sometimes they cook the aborted fetus so that its better for the dogs and sometimes they cut it up and give the dogs raw.

Respondent: Its cut into pieces because we often have many dogs and you need to share out the meat otherwise some weaker dogs may not get a share.

Respondent: On the other hand, if you give the meat to the calf without cutting it into pieces then the dogs might form the habit of eating healthy calves(laughter). If you cut it then you give it to the dogs yourself but if you leave them to pick it themselves then the dogs might form the habit of catching and eating new born calves while the mother is still lying down after birth. If the fetus is too small like 2 or 3 months, then we just leave it to the dogs to eat it. The dogs just pick it up because the herds boys usually go to graze cattle accompanied by dogs.

Q8 Perceptions on infertility in cattle

Interviewer: What causes infertility in livestock?

Respondent: We don't understand…we just say the cow is infertile.

Respondent: Each time it is mating…it mates constantly. It doesn't conceive so you sell it. Also you can keep it and slaughter it for people to eat meat when you have a function. And that's it. We don't keep it and we don't know why it is infertile.

Respondent: Let me ask you, what causes infertility in cattle?

Interviewer: Brucellosis is one of the causes. They asked other symptoms, treatment.

Q9 Perceptions on reduced milk production

Interviewer: What causes reduced milk production?

Respondent: Its because of trypanasomiasis and once the cow is treated it recovers and milk production resumes.

Q10 Perceptions on hygromas

Interviewer: What causes joint swellings (hygromas)?

Respondent: (Pause)…we have never seen that.

Q11 Perceptions on retained placenta

Interviewer: What about in the case of retained placenta?

Respondent: I have never seen that.

Respondent: The men treat the cow using herbs and feed the cow and the placenta comes off. They also use conventional medicines and it comes off.

Q12 Perceptions on weak or still born calf

Interviewer: Why do you think causes a cow to give birth to a weak or still born calf?

Respondent: We really know why. We just say that the reason is because the cow was sick and thus the still born or weak calf. That is what we think that the cow passed off the illness to the calf.

Respondent: So we treat the cow itself. We just see men treating the cow but we don't know for what because we don't ask.

Q13 Common human diseases in this locality

Interviewer: What are the common human diseases in this area?

Respondent: TB, Malaria

Respondent: Lower backache, hip pain and leg pains. According to me these are the most common illnesses among women these days. A lot of women are going to hospital because of these conditions. I don't understand why. Some say its because of the use of contraceptives but some women are not using contraceptives and they are still afflicted.

Respondent: That's true for some people but not all.

Respondent: UTIs.

Respondent: Typhoid too.

Respondent: Yes, every time you go to hospital you are diagnosed with typhoid and UTI and malaria.

Respondent: UTI and typhoid and malaria.

Q14 Signs of a febrile illness

Interviewer: What are the symptoms of a febrile illness?

Respondent: Feeling cold so you want to be in the sun all the time, headache, fever is the main symptom, dizziness,

Respondent: Body aches and fever

Q15 Treatment pathways related to febrile illness

Interviewer: What is the first course of action that you take when you feel these symptoms above?

Respondent: We go to the hospital…. first we take Panadol and if not feeling better you go to the health facility where they conduct tests like those for malaria and UTI and then you get treatment.

Respondent: I will first take Panadol and then if don't feel better I will go to the hospital.

Interviewer: Do you use herbal medicine?

Respondent: Yes, some use especially for stomach pain. Especially if the pain comes on suddenly. And if you don't feel better you know its malaria and so you go to the hospital.

Respondent: Usually its adults who use herbal medicine. They use them because it's a practice they have inherited from the forefathers.

Respondent: Yes, we use herbal medicines but usually for stomach pains only but not for febrile illnesses.

Interviewer: What about children, do they use herbal medicine?

Respondent: No. We take children to the hospital because children are not able to fully express how they feel so they might be severely ill. For a child you cannot really be sure what they are suffering from.

Interviewer: What happens when you go to a health facility?

Respondent: They will test to see what you are suffering from and then give you medication and if they don't have the medicine they ask you to go buy it.

Respondent: The clinician will conduct the necessary tests for typhoid and UTI and then give me the right medication. However, here in Sagamaganga they only test for malaria so the other tests you get them in the Lab in Lungongole or at Kibaoni.

Interviewer: Are there times you take the medication provided and still not get better?

Respondent: Yes, it happens. And in that case you go to KIbaoni where they have better tests because here at Sagamaganga they don't any facilities to test for anything.

Interviewer: You said earlier that they test before giving treatment. How do they do that if they have no facilities?

Respondent: They don’t test stool and urine.

Respondent: They don’t test, only for malaria.

Interviewer: How then are you diagnosed with UTI or typhoid?

Respondent: When you are suffering form malaria they treat you but if its not malaria they send you for further tests at Kibaoni.

Respondent: They test and treat at Kibaoni.

Respondent: UTI…I don't know where it has come from. I only hear about it. But it is very common these days,

Interviewer: So the only test they perform here at the Sagamaganga health Centre is malaria?

Respondent: Yes, and if they find that you are not suffering from malaria they refer you to Kibaoni Health Centre or to another lab.

Respondent: They also refer us to the private lab at Lungongole and get tested for malaria, UTI, typhoid…(pause) and if you don't get better after the treatment you've bought you go to Kibaoni where they have more tests.

Interviewer: Please help me understand what goes on once you've gone to the lab?

Respondent: The lab technician advices you which tests to do and then after doing them he will sell you the appropriate medicines.

Respondent: Let me ask. What is the appropriate drug for UTI?

Interviewer: There are different medications for treating UTI….

Respondent: Am asking because we are given drugs but we don't get better and when we ask they tell us you must get an injection first.

Respondent: This disease is so stubborn to treat, even men keep talking about it. Now it is being treated using injections and men are injecting themselves but they don't involve their wives. And when you ask them they tell you that it's a disease they are suffering from but they wont disclose which one. And when we go to the health facilities they advice us to obtain UTI treatment alongside our husbands and co wives but they don't want to involve us when they are getting the treatment.

Respondent: Those men who are injecting themselves only are fools because if they don't involve the wives they keep getting re infected.

Respondent: And with UTI one gets very bad stomach pains. This disease is so troublesome. Eh, UTI! We wonder what is it?!

Interviewer: Was UTI not common in the past?(very animated discussion and laughter)

Respondent: They have just changed the name of the disease. This disease has a Kisukuma name(laughter). We call it "kaswende"

Respondent: And with this disease if you delay in going to the hospital you are not able to conceive. This is the same disease.

Respondent: There is "kaswende" and "kasokane". And both of these diseases are treatable using herbal medicine but with hospital medicines you will not get any correct treatment. You don't get better.

Interviewer: Do you involve both partners for the treatment of "kaswende" and "kasokane"?

Respondent: Yes, with the herbal medicine for these two illnesses both husbands and wives were treated.

Interviewer: Why is it then that the husbands don't want the involvement of their wives now?

Respondent: I don't know. Maybe they are shy.

Respondent: And we are told to abstain from sex during the course of the treatment. But some men don't heed the advice.

Respondent: But I really wonder if it really is UTI or just some bacteria that infest the tummy. Then they just claim its UTI..

Respondent: As she said we really don't understand. For example, even a toddler of about 2 years old is diagnosed with UTI. How can a child get UTI at 2 or 3 or 5 years od age.

Respondent: This disease is common during the heavy rains because we are consuming the dirty rain water and so even children get it.

Respondent: Yes, if they say its because of the dirty water then I can believe that.

Respondent: Another cause of UTI is the pit latrines that we use in our homes because the urine splashes on you as a woman when you urinate(laughter). There are many ways of catching this disease.

Interviewer: Are you saying then that the commonest febrile illnesses here are Malaria, UTIs and Typhoid?

Respondent: Yes

Interviewer: Do people here buy over the counter drugs without a prescription if sick?

Respondent: Oh yes, they do. Even for babies and children they do buy especially malaria drugs “mseto”. This is a form of first aid. We do it for stomach pain too.

Respondent: We do this because we buy those medications and we feel better. Even children do. So we only go to the hospital if the patients condition is not improving. And we tell the clinician that the medication we were already taking. So then they will get tested for malaria and others and given the right medication. So the over the counter drugs are mainly used as first aid.

Respondent: How do little kids get UTI because married couples infect each other…how then do kids get it?

Respondent: The clinicians tell us that there are several ways of getting UTI. One of them is that here are bacteria that reside in flood water and once we use that water we get UTI and the disease likes to be in the private parts. So this disease can be caught by anybody, children included because there are many ways through which you can catch it.

Respondent: Men do not accept to go to the hospital with their wives for the treatment of UTI because they claim to be fine and they actually do look well. And when we go to Kibaoni its true we find that “everyone” is suffering from UTI. Both men and women. I thought previously that we get UTI from drinking dirty water. (long discussion about “mkojo mchafu” in Kisukuma).

Interviewer: Please interpret for me what you all were talking about?

Respondent: I want to know, UTI comes from drinking dirty water? Is that the case?

Interviewer: It comes from bacteria introduced to the urinary tract system like through wiping back front after long call.

Interviewer: Do people in this locality conclude that they are suffering from a certain illness from the symptoms they are experiencing?

Respondent: Yes if it is malaria I know..i know when I am suffering from a febrile illness

Interviewer: Malaria or febrile illness?

Respondent: "Malaria ni homa na homa ni malaria"= Malaria is febrile illness and febrile illness is malaria. So if I feel cold I know it is malaria.

Respondent: Also headache and stomachache are all signs of malaria

Respondent: Bodily pains and vomiting are also signs of malaria

Interviewer: How about typhoid?

Respondent: (Pause). This one is hard to know.

Interviewer: What about UTI?

Respondent: Also you can't tell

Respondent: You can because as you finish to urinate there is a lot of pain

Interviewer: Are there instances after this identification that people go and buy over the counter medications without going to a hospital?

Respondent: Yes, some do based on how they are feeling. But some get tested first.

Respondent: If they don't get better then they go to the laboratory or the health facility in Sagamaganga. If not better, they go to Kibaoni and finally if still unwell to St Francis where they have elaborate tests. This can quite a while.

Interviewer: Are health services easily accessible?

Respondent: Yes, they are but it takes a long time before you are served. You could even go to the health facility whilst sick and spend a whole day there conducting tests and still not get any treatment. You might have to go back the following day. The queues are usually so so long! And also if very sick patients come they are allowed to jump the queue and be seen by the doctor since their condition is dire. That is why we buy over the counter medicine because we queue for so long at Kibaoni. And God helps us and we recover. Its so much trouble going to Kibaoni and costly because if you have to go for two consecutive days you spend Tsh 12000 since one-way trip is Tsh 3,000. And you need to include money to buy lunch too. So we first try over the counter medication and usually we get better. It would be much better if we had a proper lab facility in our dispensary here in Sagamaganga. The lab in Lungongole is private and pricey too so if you don't have the cash you go to Kibaoni and incur the trouble of cost and waiting. Some tests in Lungongole cost Tsh 7,000. Its too expensive since it's a business and money making venture.

FGD Lungongole Women

9/8/2019

Q1 Gender roles in livestock management

Interviewer: Why do you keep livestock?

Respondent: For us the Wasukuma we don’t have any other means of making a livelihood so we have to keep livestock so as to meet our livelihood needs. This means we depend on livestock to educate our children for example. Also in constructing a house. We don’t know how to do business so we depend on livestock.

Respondent: Also livestock enable us to buy food.

Respondent: Cattle also gives us milk.

Respondent: Cattle is our bank (Laughter). One of my children was educated in Mwanza at TSH 4,000,000 so we sold cattle to educate him.

Interviewer: Please tell me why you keep goats, cattle and sheep?

Respondent: Goats are only used to cater for small financial needs since you cannot sell them for much in the market.

Respondent: Goats also do not give us milk too. Only those who have chest illnesses take goat milk here(laughter).

Respondent: We also slaughter goats when we have a visitor or when we call people to help us with farm work. We slaughter a goat for meat.

Interviewer: Please tell me about the division of labor related to livestock production by gender?

Respondent: We all are involved in milking. Herding is done by men and women are only involved if men are unavailable like busy in the farms or unwell. Treating of sick animals is done by men. Preparation of skin and hides by men and assisting in parturition is done by both men and women. In general men do most of the livestock related duties and women do household chores, look after children and participate in farm work.

Q2 Practices on assisting with parturition

Interviewer: Are gloves used when assisting cows during parturition?

Respondent: No we don't use gloves. We use our bare hands.

Interviewer: Do you know that it is safe to use gloves?

Respondent: Yes, we do but where do we get the gloves? (laughter). If a cow is giving birth in the pasture areas will you first run to the shopping center to buy the gloves (laughter). I just help my cow and then go wash hands with soap and water. Even the young men who look after livestock also wash their hands after assisting a cow.

Q3 Practices on residing with livestock

Interviewer: Do humans reside with livestock?

Respondent: That used to happen in the past not now. Nowadays people build separate dwellings for livestock.

Respondent: Is there any harm in livestock and people sleeping in the same area? (laughter)

Interviewer: Yes, it can be dangerous. Explained about aerosol transmission of brucellosis.

Respondent: People these days don't dwell with livestock. Its mainly because the livestock make a house very dirty and these days we do not want that dirt. In the past people were not so careful about that because we also slept on very high beds and goats would under the bed. These days' people sleep on proper beds and mattresses so we don't keep livestock in the house.

Q4 Perceptions on raw milk consumption, boiling milk and community engagement

Interviewer: Please tell me about the consumption of raw milk in this area?

Respondent: Usually we just milk and then ferment the raw milk after sieving it. Then once the milk is fermented we remove the milk fat (samuli) and then we drink the fermented milk. We have heard people saying that it they like boiled milk but most of us grew up drinking raw milk so its not our habit to boil milk.

Interviewer: Do you know that it is important to boil milk though?

Respondent: Yes, we do because they say that once you boil milk it is very good for consumption. However, because most of us are not used to boiling milk we are unable to drink it. We cannot stand the taste. (laughter). The milk is not pleasing at all once it been boiled.

Respondent: The milk acquires a different unpleasant smell once its been boiled and so we don't like it. Raw milk is a lot better, we are used to raw milk and are not accustomed to boiled milk.

Respondent: For us the Wasukuma once a child has been weaned and is old enough, in the morning the child will pick a cup and head to the cattle shed and get milk as it is being milked. And the child becomes big and grows very well. The milk has no harm at all. No harm at all. We don't want boiled milk at all. We milk and immediately give it to the child while its warm from the cow's udder.

Respondent: I have ever heard that raw milk is dangerous but I don't know what harm is in the raw milk. I have only heard that from some women around but I don't know what danger.

Interviewer: After you heard that raw milk is dangerous did you start boiling milk?

Respondent: Oh, no (laughter). We are used to raw milk, its our culture. We are used to raw milk.

Interviewer: Do clinicians advice you to boil milk before consumption or in the radios?

Respondent: Yes, they tell us but we don't take that information seriously. We just drink our raw milk (laughter) while it is warm from the cow's udder and that is very nice.

Respondent: (Older woman) Since I was born I have taken raw milk all my life and I have never needed to have a blood transfusion or to be rehydrated in hospital (laughter) and I have never been admitted in a hospital too so I don't know what have milk can possibly have.

Respondent: They even ask us to boil milk before fermenting it but we don't see any harm ourselves in not boiling the milk.

Interviewer: Did people here boil milk during the last RVF outbreak?

Respondent: Yes, we heard them announcing that we boil milk. Others boiled but others did not. Yes, they told us but most of us did not.

Interviewer: It appears that most of you here have heard that it is safer to boil milk before consumption. What do you feel about that?

Respondent: Please clarify to us why we need to boil milk first.

Interviewer: Explained to them the need to boil milk to reduce the risk for certain zoonotic diseases.

Respondent: Most of us have heard about boiling milk but we don't have a clear understanding of the risks involved.

Interviewer: Do you feel that if people had a clearer understanding then that they would start to boil milk?

Respondent: (pause) We can boil milk if we know what diseases can arise form raw milk consumption.

Respondent: On the other hand, we have never come across anyone who became ill as a result of raw milk consumption. We are used to consuming raw milk.

Q5 Practices on herding and attitudes of herdsmen

Interviewer: What age group does which livestock related duties?

Respondent: From the age of five young boys go and herd the kids and lambs. By 6 and 7 years of age they are able to leave and go herding all day and they look after calves two by two. 9 and 10 year olds are able to look after cattle. However, these days' children go to school so those who heard are usually older. Like 20 year olds go to the grazing area to look after livestock. And at that age they are able to treat sick cattle and perform all livestock related duties.

Interviewer: When do they go to the grazing area to live there with the cattle for pasture?

Respondent: They go and live there until the area starts to flood. They go in June until end of March or early April.

Q6 Livestock wildlife interaction

Interviewer: Do the livestock interact with wild animals in the grazing area?

Respondent: Yes, there are wild animals. Various kinds of animals are there like lions, buffaloes, elephants, “sheshe”. They definitely interact.

Interviewer: Do you think that there is any harm in livestock and wildlife interacting?

Respondent: Yeah there is harm and that is why we treat the cattle once they are back from the grazing area. If we don’t treat them then they get sick and have abortions. This is because of “Ndorobo” which bite buffaloes and later bite cows and after that the cow does get affected and it leads to abortions.

Interviewer: So in that case then you treat the livestock?

Respondent: Yes, we treat the livestock for “Ndorobo”.

Interviewer: Is there any other harm in this interaction?

Respondent: None at all.

Respondent: I want to ask. Lions also prey on livestock. Crocodiles also eat our cattle when the cows are taken for watering close to the Kilombero river.

Q7 Common livestock diseases and practices related to abortions in cattle

Interviewer: What are the common livestock diseases in this area?

Respondent: (pause). We only know “Ndorobo”, worms and CBPP.

Interviewer: How do you identify a sick cow as women?

Respondent: Usually we go to the cow shed each morning and we are able to tell a sick cow. This is when we notice a rough hair coat, running nose, panting and drooling.

Interviewer: Are you able to suspect what disease the cow is suffering from?

Respondent: Not really

Respondent: With CBPP, the cow will be coughing and panting. And so you treat the cow with an injection. CBPP is present all the time.

Respondent: With Lusoona (FMD) the cow will have sores in the mouth and hooves. This disease is common during flooding. “Ndorobo” is also present all the time.

Interviewer: Are you familiar with any diseases that are transmitted from livestock to humans?

Respondent: (PAUSE). I am not familiar with any.

Respondent: Yes, tetanus only.

Interviewer: Have you ever heard of Rift valley fever?

Respondent: We have ever heard in the radio but we don’t have good information. We heard that it is from some other countries and we should not eat meat. Some stopped but went back to eating soon after.

Respondent: The RVF outbreak was in Dodoma but not here but they told us not to eat meat even here and we stopped for a while. However, we went back to eating the meat because good healthy cows were dying and so we would roast the meat and eat. (laughter). It was tough to waste meat. We would slaughter the fat ones and only discard the cows that looked very weak and emaciated because their meat would be slimy too. Even the butcheries were closed and they did not sell meat in that season. But the healthy looking ones we would dry the meat and then preserve it after it is well dry. Then we would store the meat and only remove the portion required for a meal and boil it then stew it.

Interviewer: Did you witness any cases of RVF in humans in this area?

Respondent: None at all. We were not cooking the meat while its fresh we were drying it over a fire first.

Respondent: Even fresh meat we cooked too. The drying was to preserve the meat to avoid spoilage.

Interviewer: How do you generally dispose off a cow that has recently died?

Respondent: It is often buried.

Respondent: However, if the cow is "good" and healthy looking we slaughter it and eat it. (laughter). We only give dogs the internal organs like liver and lungs if they look diseased and then we eat the rest of the meat. We cannot throw away meat. We have to access the internal organs like intestines, stomach, liver and lungs and if they look suspicious those we throw away (give to the dogs) but not the meat.

Interviewer: Have you ever heard of anthrax?

Respondent: Yes, but I don't know if it is transmitted to people as well.

Interviewer: Have you ever heard of the disease brucellosis?

Respondent: (Pause) Ah....(they discuss among themselves)

Respondent: What are the signs of brucellosis in livestock?

Interviewer: I respond and explain brucellosis symptoms in livestock.

Respondent: We have never heard of it. And in the health facilities they don't mention it also. It is always malaria, UTI or typhoid that they tell us a lot about.

Q8 Perceptions on infertility in cattle

Interviewer: What causes infertility in livestock?

Respondent: We see them but what we do is we use the infertile cow as a draught animal and then the cow loses weight and then it is able to conceive.

Respondent: The cow is infertile because its too big and fat. If the cow is infertile and doesn't conceive even after being used as a draught animal, we sell it for meat. We can't continue staying with a cow that is not reproducing. Those cows become so big and so they fetch good prices in the market.

Q9 Perceptions on reduced milk production

Interviewer: What leads to reduced milk production in cattle?

Respondent: (pause). This is caused by a cow that is not feeding well and has been watered in a dirty well. But if a cow is feeding well and taking clean water then it will produce adequate milk.

Q11 Perceptions on retained placenta

Interviewer: What do you think causes retained placenta in livestock?

Respondent: We have a local herbal drug that we feed to the cows and the placenta comes off. It is never a big problem. Once we use that medicine the placenta comes out even after a number of days.

Q12 Perceptions on weak and still born calves

Interviewer: What causes the birth of weal or still born calves?

Respondent: (Pause) We don't understand why that happens.

Respondent: Maybe it is because the cow has some illness.

Q13 Common human diseases in this locality

Interviewer: What are the common human diseases in this locality?

Respondent: The first is aches and pains in the legs from the knees going down especially women.

Respondent: Also typhoid, UTI and malaria.

Q14 Signs of febrile illness

Interviewer: What are the signs of a febrile illness?

Respondent: Feeling cold, headache, flu like symptom, fatigue, fever, stomachache.

Q15 Treatment pathways related to febrile illness

Interviewer: What is the first step you take when you have the above symptoms?

Respondent: We go and buy Panadol to reduce the pain as you think of the next course of action. Then go to a laboratory the following day to do some tests. There is a lab here in Lungongole or in the mission hospital in Kikwawila.

Respondent: After using Panadol then you buy malaria medicine and only go to the hospital if you take this and still don't feel better. You buy the malaria medicine because the first disease we suspect is malaria because it is very common here. And sometimes we get better after these drugs.

Interviewer: What kinds of tests do they do in these laboratories?

Respondent: They conduct tests for malaria, UTI and stomach ulcers. Each tests has its own cost. The attendant advices you on the tests to conduct based on your symptoms. It's not the same as going to a clinician all the do is give you advice. Also you can propose the kinds of tests you would like him or her to do as a client.

Interviewer: What's the difference between visiting this private lab and a lab in the hospital?

Respondent: In the hospital the clinician advices you on the test to conduct. But in the private lab facility you can suggest the kinds of tests you need done like malaria or typhoid. Then you go buy the medicine recommended.

Interviewer: Do you take the same steps when children are sick with a fever too or you do it differently?

Respondent: For children we do the same especially if they get ill at night. We start with Panadol because we can't take them to the hospital at night. Then in the morning if they are still sick then you take them to the hospital.

Interviewer: Do you take children to have tests done in the private facilities too?

Respondent: Yes, we do because even if we go to the government run health facility the child will still only be given Panadol and malaria drugs. They don't do any tests so we just prefer to take them to the lab here. They only touch the child's forehead and then prescribe Panadol and malaria drugs. They use their hands and then ask a few questions like "is the child vomiting?", "did the child have a fever at night?", "is the child coughing?". They do this as they touch the child..they use the "hand test" (laughter). Then they tell you the child has sores in the chest, how do they know that just by touching the child? They don't do any tests in these Government facilities for sure. They just prescribe Panadol and malaria drugs. So you are better of in the lab so that they can test and give you the proper diagnosis. In the government facility you queue for so long and all you get at the end is Panadol and a prescription to go and buy malaria drugs. And sometimes this doesn't help because the child doesn't recover and you have to go back to seek medication.

Respondent: That is the case for even adults. They just ask questions and then prescribe. They don't do any tests. They only prescribe without tests. Only on very few occasions do they test or those who are gravely ill and need admission. For the rest of us they just use their hands and then prescribe.

Interviewer: What kinds of medicine are commonly prescribed?

Respondent: Usually malaria drugs, drugs to treat UTI if you complain of lower abdominal pain and that is why most of us go to private heath facilities. In the government facilities, we queue all day and no tests are done but in the private facilities they are prompt and conduct all the necessary tests.

Interviewer: Does this apply to the referral hospitals too like Kibaoni and St Francis or only the dispensary in Sagamaganga?

Respondent: Hey! If one goes to Kibaoni you'll be there all day and you'll leave without medication and have to go back the following day to get the results of your tests and to see the clinician for medication. You take two days and its costly because you need at least Tsh 6,000 for transport not including money to buy lunch. St Francis is a bit better because you pay for the services but Kibaoni they charge very little.

Respondent: I was in Kibaoni two days ago because of toothache. It was a Saturday and they sent me home without any medication not even Panadol. So I went home and used herbal medicine and this relieved my pain and I have not gone back to the hospital.

Interviewer: Which private hospitals do you go to?

Respondent: Usually hospitals in Ifakara or the mission hospital in Kikwawila. In the private hospitals the required tests are done and you are attended to fast. There is no delay.

Interviewer: Do people use herbal medicines for febrile illnesses?

Respondent: In the past we did but not any more.

Respondent: We still use herbal medicines for throat sores, toothache, stomach ache, back ache. But with febrile illness you have to visit a health facility.

Respondent: Herbal medicine was used in the past not nowadays. However, herbal medicine is used for women who find it hard to conceive and they do (laughter) even after hospitals fail to treat the infertility.

Interviewer: What are your recommendations for improved health care in this area?

Respondent: That the clinicians in the health facilities attend promptly to patients, sometimes even children are not attended to promptly. We need a health facility here in Lungongole because the closest government facility is in another village Sagamaganga.

Respondent: If you don't have cash you cannot access services in the private lab facility. Plus, it is closed by 6 pm. In Kibaoni children are attended to promptly but not adults. Most of the health facilities also do not stock any drugs so they ask you to go buy the medicines in the private chemists which are owned by the same clinicians. Only children are given medication for free.

Respondent: You must go to the hospital with enough money because all the services will cost something. No clinician will attend to you without the needed cash. They do not attend to you. Even if you have insurance it does not help, it will only cater for Panadol and not any other medication. The insurance does not help us at all. If you pay cash, you move faster but if you are using insurance you queue for even longer.

Respondent: And most of us are often only diagnosed with UTI, malaria or typhoid. Like the disease you mentioned, brucellosis I have never even heard of it. Not even once.

Interviewer: Have you ever encountered individuals who suffer from febrile illnesses repeatedly? What cause of action do they take?

Respondent: They just keep going to the hospital and if they still are not well then they now start seeking herbal medicine from traditional practitioners. Sometimes, they get well. They seek another alternative. We go to these people once we don't get any cure from the hospital after many visits.

Interviewer: It appears that there are challenges to obtaining good treatment in the government health facilities.

Respondent: I was once admitted at Kibaoni and then the nurse told me to go buy some medication. Then some inspectors came and asked me if I had been asked to go buy medication and apparently the medication was available in the hospital. Another patient was being treated with the wrong medicine. The nurse started bribing me with Tsh 10000 so that I don't disclose what I had been told. I took the cash and kept quiet. There is a lot of corruption in the health facilities, like Kibaoni. It's a big problem and that is why we prefer the private health facilities because they are kind and loving.

Respondent: Even with child birth it is a big problem. You just trust God to help you. The nurses are unkind and insult us. Some women give birth on the floor while nurses are on their smart phones. They insult us especially us the Wasukuma women as they tell us we give birth to many children. Then they ask for some Tsh 5000 or Tsh 10000 because apparently they have helped us. And yet it is their job to help us in child birth. They demand money from us. They are greedy and selfish. Most of these nurses are women but the male nurses are usually very helpful. We are very happy when we find male nurses. The female burses claim that they helped to prevent hemorrhage for example and need to be paid by us. These things are very common in Ifakara and not in other areas. We feel helpless because if you are alone in hospital what do you do but to cooperate with the nurses?

Respondent: Some of us even bribe the clinicians so that they can get prompt treatment while others are on the queue. We are our own enemies because we are the ones that give those bribes too. Why should we pay them for doing what they are supposed to do?

FGD Men Siginali August 2019

Q1 Gender roles in livestock management

Interviewer: Who looks after livestock? Milking, treating, herding

Respondent: That is men's work. Women work at home like milking but most duties belong to men.

Interviewer: At what age do young boys start to go out to look after livestock in the grazing area?

Respondent: From 8 years of age all the way to 30 years of age. You know the grazing area is laden with wild animals and so mature people are needed there. The younger boys go to look after calves but the older cattle are taken care of by the older boys and men.

Q2 Practices on assisiting with parturition

Interviewer: Do you ever wear gloves during assisted parturition?

Respondent: No we use our bare hands.

Interviewer: Have you ever heard that it is important to use gloves?

Respondent: No, we don't have experts to educate us.

Respondent: They are not available.

Respondent: We are used to assisting cattle with our bare hands.

Interviewer: Do you think it is dangerous to assist cattle to give birth using bare hands?

Respondent: There is no danger. If you as a herdsman needs to assist your cow and you are in the wild where do you find gloves?

Respondent: Also if it was dangerous the calf would also be harmed.

Interviewer: If gloves were to be provided or available would you use them?

Respondent: Yes, we would use them. We would use them to avoid being dirty.

Respondent: All these things are good like I said earlier but we need proper education and sensitization. Most people do not know and they have been doing it for many years with no harm to them (laughter). If you tell this old man here that it is harmful, he finds it interesting considering he has assisted cattle for years without suffering any harm. Its like how women used to be told to use mosquito nets and they would just store them and not use them. So education is very important. Like I told you earlier we need engagement with the experts where we do things practically. We milk a cow then discuss what kinds of bacteria are in milk. We need to see using machines to actually see the bacteria and see them. If I see the bacteria, then I will become the teacher myself. Even with HIV many people did not believe it was there until they were tested and found to have it then they believed and yet they were just taking panadols. So I request that you organize practical education for us so that we can actually see that there is bacteria. So we compare raw to boiled milk and you show us the bacteria in milk (laughter).

Q4 Perceptions on raw milk consumption, boiling milk and community engagement

Interviewer: Please tell me more about the consumption of raw milk in this locality

Respondent: We prefer raw milk because it is our practice from long ago. We inherited this practice from our forefathers.

Respondent: Raw milk is preferable because all the nutrients are in it and it has protective value. Even when we are exposed to various kinds of harm In the wild while we herd cows we are safe. Actually we have realized that even as we engage in farming and the dust from rice farming does not harm us. You find that as we harvest rice we consume raw milk so that the dust form the rice doesn't affect us negatively. On the other hand, we walk amidst a lot of cows and its very dusty and the dust doesn't affect us because we consume raw milk. Also once you take raw milk you can go for up to two days without feeling hungry, all you need to drink is milk. That is why we do not boil milk because raw milk has a lot of important nutrients and makes us very strong.

Interviewer: So you are saying that once you boil milk you kill essential nutrients?

Respondent: Yes, once you do that you "kill the milk" and the milk becomes…water is even preferable to boiled milk because the latter has no value at all. It is dead.

Respondent: Raw milk also cures stomach ulcers. Once you boil the milk then you remove the fat in the milk which is what kills the organisms that cause stomach ulcers. Even when you consume poison and then consume raw milk then the poison is neutralized and it doesn't harm you. So raw milk is like medicine.

Interviewer: Have you ever heard of the importance of boiling milk?

Respondent: Yes, I have from radio and they say it is important to boil milk because there are bacteria that can be found in the cows' teats and thus transferred into the milk. And also as cows feed the they also get certain bacteria from the grass that can lead to human disease. Also if the person milking the cow is not hygienic then the milk can harm people.

Respondent: All of us here have heard that we need to boil milk. A lot of experts tell us to boil milk but we are not used to boiling milk.

Respondent: We are used to consuming raw milk. The only solution is to provide us with regular education until we get used to boiling milk.

Respondent: Also we know that our forefathers lived long and they were consuming raw milk. But these days there are a lot of diseases because of climatic changes. For instance, our forefathers did not go to hospital and they were healthy. They only took traditional herbs. In the past we didn't hear of people who had anemia or dehydrated like we do these days. That is why we don't like to boil milk. So right now we need to be educated.

Respondent: We need regular education like twice a week or several times in a month especially for the women because they are the ones that cook. If women change then the households will be different. For us men, we will accept whatever we are brought to eat.

Interviewer: I hear you. What is the best way to educate agro pastoralists? How can we best reach them?

Respondent: Through the radio is a good way but we don't have electricity here so it's a challenge but still its better as you reach a lot of people. Advertisements are a good idea through the radio.

Respondent: That is okay but I don't think it's the best way because most of us have not been to school so they may not understand the message. The kind of education we need is practical where the livestock keepers can engage with scientists and policy makers. Through radio is okay but some do not have radios or they don't listen to them. Others are in the wild with no radio access too so they can't know. But if experts came here and they engaged us and we discussed then that might work. Actually in our experience those who live in the wild and look after livestock are healthier than we are. Those who stay in the wild and they don't even bathe and they consume raw milk they are hardly ever sick unlike us who live at home. So when we are told raw milk is harmful we fail to understand. Those live very wild lives, they don't shower, they don't use mosquito nets and they are well.

Respondent: So as you say we can get infected through raw milk. So is the raw milk still harmful even if we ferment the milk?

Interviewer: No the raw fermented milk is still harmful to people for sure.

Respondent: What are the signs of brucellosis in humans?

Interviewer: It’s a febrile illness and usually misdiagnosed so most people become of ill health.

Respondent: Maybe we are already infected because as you can see we all are dozing off!

Interviewer: Do women listen to the radios?

Respondent: Yes, some do. I think this is really a women’s issue in terms of educating women and this need to be especially done in the pre natal clinics and women need to be taught there. The same way they teach women about proper nutrition for babies or the use of mosquito nets. The same needs to be done in regards to milk consumption.

Q6 Livestock wildlife interaction

Interviewer: What kinds of wild animals are in that area?

Respondent: There are lots of animals because of Selous game reserve. Some wild animals come from Selous to the grazing area. These animals include lions, crocodiles, buffaloes are there too but these ones do not harm cattle because they belong to the same family. These ones are not harmful to cattle only harmful to humans. There are also antelopes.

Interviewer: Is there any danger in cattle grazing together with wild animals like buffaloes and antelopes?

Respondent: Aaaaah…(pause). For now, there are no issues…its very rare that buffaloes cause harm to human beings…very occasionally. But animals that have claws are constantly harming our cattle.

Respondent: Yes, those cases occur where livestock are harmed by wild animals and even occasionally when they harm people. However, there are also instances where cattle are harassed by people when they encroach into game areas. However, when buffaloes come to the grazing areas that is not considered a problem. And when buffaloes feed on the grass in the grazing area there really is np problem other than the grass being depleted. When it happens that cattle get into the game areas then they are confiscated and the owner fined. Now those are our challenges. One animal is favored over the other one.

Respondent: Yes, it's a criminal trespass when cattle get into the game areas but not criminal if the buffaloes encroach into the grazing area. The grazing area borders both Selous and ILUMA. You get into ILUMA first before you get to Selous.

Respondent: So when cattle get into ILUMA and Selous it's a trespass because apparently they are destroying the ecosystem but when buffaloes get into the grazing area its not considered a trespass or a destruction of the ecosystem. And yet the hooves are the same. It's a kind of harassment because both animals have the same effect but the cattle we raise are considered more destructive.

Respondent: If you observe keenly actually, “sheshe” antelopes have the same kind of hooves as goats and buffaloes as cattle. But now as we said it’s a problem and we are really harassed even beaten. The thing they should do is to put a barrier between the two areas so there is no trespass. Or if they find that wild animals got into the grazing area then the game department should also compensate the livestock keepers as well. So it should be 50/50 cattle and wild animals should be given the same treatment.

Respondent: There are fines both ways when a farmer encroaches into the grazing area to farm and vise versa when livestock keepers encroaches into the farm land to graze his livestock and so it should be the same between wild animals and cattle. As it is when cattle get into the game areas the livestock keepers are fined but not when buffaloes get into the grazing area. So Is that just? So we feel that we are considered as trespassers when our cattle get into the game areas but not when the buffaloes get into our grazing area. That is not fair. Even right now if we went to the grazing area we would encounter (“sheshe”) puku and buffaloes and they are not harmful.

Respondent: However, these wild animals deplete the grass for cattle as well as water because they drink from the same places. So our government in that case is not very considerate. If there was a fine for wild animals encroaching into our pastures, then we would know that there is justice. Buffaloes should also be fined; the Government has hired people to look after buffaloes just like we look after cattle. The government should also pay the fines. When w plan for land use each area should be respected and not some and some not.

Respondent: But if we go to the game areas with our cattle we are hunted down with guns. And wild animals are many. As you go to water your cattle you meet with “sheshe”, crocodile’ and hippos. And when these animals eat cattle it is not considered a criminal act but if cattle eat grass in a game reserve it is considered a criminal offense. So they claim that livestock keepers are criminals and one is fined heavily at 50,000Tsh per cow. If you have 500 heads of cattle how much money do you need to pay the fine? And you will often be apprehended almost ten times each year. O think they want to confiscate all our cattle and impoverish us and then relocate us form here to a place we don’t know. And you know this is like a business to the game wardens. Instead of looking after the wild animals they are often at the border with our grazing area looking for any trespassers. If they see any cattle nearby they run after the herder and when he flees the game wardens drive the cattle into the park. However, these days it’s a lot better and the smart phones are very helpful. In the past the herders were really being harassed but we had a meeting with the District Commissioner and he told the herdsmen to be taking pictures of the wardens as they drive the cattle into the game reserves as evidence in their favor. And this has helped somewhat. But its still a problem because the wardens have guns and they intimidate us with the guns. And you know they have cattle holding pens in the park where they keep the cattle that has been apprehended and wait for fines or bribes to be paid.

Interviewer: So phones have helped?

Respondent: Yes, but still if they notice that you are taking pictures your phone will be forcibly taken and destroyed. So you have to take the pictures secretly.

Respondent: Its so hard here because if you look at Ngorongoro game reserve humans, livestock and people co exist peacefully. How come they don't have as many restrictions as we do here and yet our game reserve is smaller? Why are they so unfair to us? In Ngorongoro, there are hospitals and schools in the park and they are also Tanzanians. Do they have a different Government? No, it's the same. We want to know how it is that they have been allowed into the park and maybe we too can get the same treatment. This would be very helpful. They would rather ask us to go live in the park and then those who misbehave like I felling trees disciplined.

Interviewer: Why do cattle get into the game areas?

Respondent: The grazing area is not sufficient. On the other hand, when it floods in the grazing area we have to move our cattle closer home where there is even lesser space for grazing. And also there is little water especially during the dry season in Sep to Nov and so we take our cattle for watering in the game reserve and that is when we are apprehended. And we actually don't get our cattle into the park they are watered at the border not inside the park itself.

Respondent: You see the cattle once they are so close to the game area they get into the park and that's when they are caught and the livestock owner is fined.

Respondent: Livestock keepers and game wardens are sworn enemies and we flee from them even if our cattle are nearby but not too close to the game areas. We run away from them because they beat us badly and we prefer that they confiscate the cattle instead. We don't want to get caught. They are armed and are many too so we are no match for them. We are like lions and cattle. So once you see them you flee even if you are in the grazing area but close to the border. You don't want to come near the game wardens at all. We cannot be friends. And when we report to the authorities they just promise to look into the issue which they never really do and each day is worse than the previous one. Its like the way parents give empty promises to their children.

Respondent: Our request to you researchers therefore is that you report these issues as they are without any editing. Report them as they are. Another one is, let this get to the president himself. He should defend all people equally.

Respondent: And usually if the government is in need of any money in the villages the people they come to is us livestock keepers for monetary contributions. Which we do but once that happens we are quickly forgotten. Once we give them the cattle or cash we are forgotten. This challenge has been present since 2012 and its not stopping. Seven years later our cattle are still being confiscated and us fined. How then can one have faith in this kind of Government that is harassing and extorting from you? Its like having an unreliable friend who only wants to benefit from you and they don't give anything in return. They are constantly asking for help from us and shortly after they confiscate our livestock. How are we to live like this and yet we depend on livestock for livelihood?

Respondent: If we lived like they do in the NCA (Ngorongoro)..they would be letting us graze our livestock in the Udzungwa National park when it floods between March and May and then we go back to our grazing area after the floods. But now they don't, they pick on cattle and act like they are not needed.

Respondent: The grazing area is only 530Ha and there are about 1,600 cattle in this village. The area is not sufficient for that number of cattle.

Interviewer: How big is ILUMA?

Respondent: ILUMA was hived off our grazing area. We used to have 2,170Ha for grazing land out of which ILUMA later got the larger share.

Q7 Common livestock diseases and practices related to abortions in cattle

Interviewer: What are the common livestock diseases in this area?

Respondent: There is CBPP, FMD, Trypanosomiasis and 'ndui" which is sores on the coat of the animal. Those are the most common. On the other hand, there are livestock officers but they never come to help with addressing these diseases. They only come to let us know that they have been posted to this area and then the disappear and come again when they need help from us the livestock keepers. In that case therefore, when our cattle are sick we treat them ourselves, we don't engage the livestock officers. When we call them they tell us to go and buy the medicine from the shops. However, when there is cattle vaccination they come because they know that there is money since we pay Tsh 1,000 per head. They are very enthusiastic when there is vaccination going on.

Interviewer: I see, what are the other challenges you face as livestock keepers?

Respondent: We have a lot of challenges for sure.

Respondent: Like they said there are flies in the wild which when they bite our livestock then they get sick.

Respondent: There are two kinds….tse tse flies and ticks. Both are a challenge. There are "Ndorobo" and "mbun'go". These are different.

Respondent: No they are not different they are the same only they are called by different names. "Ndorobo" is the Sukuma name for "mbung'o" which is Swahili.

Respondent: They are different some are bigger than others. Mbung'o are smaller and "Ndorobo" are bigger. "Ndorobo" like to stay in the wild and are big and their bite is very painful. The other smaller ones are common at the start of the rainy season.

Respondent: They are the same. The ones he's talking abut are called "naba" or "buburu". "Ndorobo" and ticks are the problem. The ones he's calling smaller are a different kind of fly which doesn't harm livestock. "Ndorobo" are the problem because even when cattle eat grass that has the eggs of the insect then they suffer from abortions too. Ticks also are a challenge.

Interviewer: Are these tse tse flies in many places?

Respondent: Yes, they especially are found in areas with wild animals and they cause abortions in cattle.

Q13 Common human diseases in this locality

Interviewer: What kinds of human diseases are common?

Respondent: The most common is malaria. Almost all the homes in this area have children taking malaria drugs at the moment.

Respondent: There are three common diseases; malaria, amoeba and typhoid. Those are the biggest challenges.

Respondent: And UTI also.

Interviewer: Have these diseases always been a challenge or just in the recent past?

Respondent: These are there all the time because we are using dirty water to wash dishes. So the water has bacteria and then when we use the utensils we get sick with UTIs. Others don't use mosquito nets often so they get malaria. Or they use torn mosquito nets. Also typhoid is a result of using dirty water and we are not used to boiling drinking water. And yet most of the water we use we get from wells and rain water and the iron sheets are dirty and dusty. The water is not safe.

Q14 Signs of a febrile illness

Interviewer: What are the signs of a febrile illness?

Respondent: Fever followed by …(pause)..its fever mainly

Respondent: And feeling cold too

Respondent: And once you have a fever then you have headache or extreme tiredness

Respondent: Joint pains too

Respondent: Stomachache is different. Malaria causes fever, fatigue then headaches or chills

Q15 Treatment pathways related to febrile illness

Interviewer: What is the first cause of action once you have a febrile illness?

Respondent: The first thing is to go to a chemist and if you find a faithful attendant he asks you to test for malaria but if he or she is not faithful they just sell you medicine.

Interviewer: What kind of medicine?

Respondent: Whichever they decide and they just administer it.

Interviewer: Can you ask that they test you?

Respondent: These people are different and some find testing you a waste of time so they just administer malaria drugs. However, if you find a faithful one they test for malaria first before giving you any medication.

Respondent: It also depends on the client because some people go and ask specifically for malaria medication. Some attendants will ask who is sick whether it's the person at the shop or someone at home and then they will give the right medication. Some people do not have enough money for both tests and medication so they prefer to just buy the drugs.

Respondent: Basically most people are not sensitized about testing and we are not used to it. For example, if I am sick I will send a friend to go buy the medication for me instead of I going to get tested. It works most of the time but its also dangerous since you might be treated for the wrong illness or take medication unnecessarily that can harm you. Like taking Panadol unnecessarily when the real problem is anemia which I am not getting treated for.

Interviewer: Why do people prefer going to the laboratories instead of to the hospital?

Respondent: First the hospital is far and thus not easily accessible. Sometimes when it floods around April the whole place is flooded and thus inaccessible. Its so hard to cross during that time. This place gets very flooded. In that case therefore they need to bring a health facility closer to us here. Because the population here is increasing and we are Tanzanians too so we need health care to be accessible.

Respondent: Secondly, sometimes the tests are also not available too.

Interviewer Are you saying then that people here go to the health facility when they are critically ill?

Respondent: Yes, its like a kind of referral hospital for us. From the laboratory to the health facility. And in that case I refer myself.

Interviewer: Do you use herbal medicines too?

Respondent: Of course we do! All the time.

Interviewer: Do you use them for febrile illnesses?

Respondent: Usually for stomach ailments and also UTIs.

Respondent I do not use hospital medicines. When I take malaria medicine I smell for up to two months. So I don't take them. If I febrile symptoms, then I take herbal medicine.

Interviewer: Do the herbs work for all febrile illnesses?

Respondent: All I know is that I get better. I don't know what they cure all I know is that once I take the herbs they work. Another thing is that, what is the value of this discussion? Is this going to help us in any way? Secondly, because you Kenyans are clever people, I wonder if you will help us in any way after this discussion?

Interviewer: The reason why we do research is to better understand the problem. I will give the findings of this research results to the Sokoine university and other policy makers in Tanzania so that they can develop appropriate strategies for the local people. I will definitely share the results of this study with the government and hopefully they can develop sustainable solutions. We also are trying to encourage people to change their habits over time.

Respondent: That is very true and we understand that.

Respondent: And like we said earlier we request that this education we requested for is brought directly to us and not left to the local authorities to deliver it to us because they will not do it. It needs to come directly to us. In the same way you need to give feedback all the way to the National level and not at the District or local level because they will not pass this information to the national policy makers.

Respondent: So please make bring us practical education. We hear for example that we should boil water for drinking but we don't. The only thing we do is occasionally heating bath water(laughter). So we really are in need of sensitization and education.

Respondent: Please tell them that we need clean water here desperately here in Kipingu. When we have access to clean water we will eliminate certain diseases for sure. And all we need are three pf so boreholes and we will be good to go. The water problems will be in the past. The wells we use we dug them ourselves.

Respondent: We were told that diseases like UTI and bilharzia come about as a result of using dirty river water to wash utensils. And that water is not safe. When this well breaks down we have to go three Km away to Mbaraji to fetch water for drinking and cooking. This therefore is a big challenge.

FGD Men Sagamaganga

18th Aug 2019

Q1 Gender roles in livestock management

Interviewer: Please tell me about the gender differentiation in livestock management

Respondent: Herding is done by men.

Respondent: Women herd once in a while when men are unavailable.

Respondent: Milking is mainly done by men and women. Among the Maasai it is the women who milk.

Respondent: Milking is also done by men and women only assist if the cows are many and there are other duties to be attended to so people need to finish milking and move on to other work. Those who milk are generally young men in their twenties.

Respondent: In our community boys start milking well enough from the age of 11.

Respondent: Men treat livestock and women can only help by holding the cow but injecting is male work.

Respondent: Women are not involved in slaughtering and skinning. It happens very rarely.

Respondent: Men also assist cows in parturition and women assist too like if the woman is looking after sheep then she can assist an ewe.

Interviewer: At what age do boys start herding livestock?

Respondent: A child at age four and five can only follow the older kids to graze livestock but cannot be entrusted with livestock to look after.

Respondent: From age 9-10 years a child can go to herd on their own is the cattle are few but more than 50 or 100 cows the boy alone cannot manage.

Q2 Practices on assisting with parturition

Interviewer: Do you use gloves when assisting cattle to deliver?

Respondent: We don’t use gloves; we use our bare hands.

Interviewer: Do you think there is any danger in using your bare hands?

Respondent: None. Or put another way we have never encountered any harm after assisting in parturition either to the animal or to ourselves.

Respondent: On the other hand, even if there are dangers we wouldn't know that they arose from that activity. Only you experts can tell us if there are any dangers in doing that.

Respondent: All am saying is that I have never encountered any harm in all my many years of assisting in parturition.

Respondent: We started doing this from childhood and we've never encountered any harm. So according to us there can be no harm because if there were we would have experienced it by now. None of us livestock keepers use gloves during assisted parturition. Sometimes we even carry newborn calves from the pasture to the home and all we do is take a shower afterwards and we've never been told in a hospital that the illness we are suffering from is a result of assisted parturition without gloves.

Q3 Perceptions on raw milk consumption, boiling milk and community engagement

Interviewer: I know most livestock keepers don't boil milk, please tell me more…would you also say that you have not experienced any harm in raw milk consumption?

Respondent: There was one time when there was a disease called RVF and during that time they boiled milk. However, after RVF was over they stopped boiling milk. They said that the cows were sick in different areas in Tanzania including here. But after the disease was over they stopped boiling milk.

Interviewer: In your opinion why do you think they stopped boiling milk?

Respondent: Because there are no diseases arising from raw milk consumption.

Interviewer: Was RVF witnessed here?

Respondent: Cattle were dying and so people were being advised to boil milk.

Interviewer: In your opinion is there any harm in taking raw milk?

Respondent: I don't think so because since we were little we have taken raw milk. They only boil for the very little ones below two years. We have never experienced any harm.

Interviewer: Have you been advised to take boiled milk only?

Respondent: We hear once in a while but when it was emphasized the most was during the RVF outbreak.

Respondent: As far as milk is concerned, experts claim that raw milk causes TB. However, there are those who boil milk but they still get TB so we don't understand how that can be then. I have a brother who had TB and was told the reason behind the TB was the consumption of raw milk. So he started boiling his milk before drinking it as he continued with medication. And then he recovered from the TB and he stopped boiling his milk and went back to raw milk consumption. This has been the case of him not boiling milk for more than 10 years and he is not sick yet they told him he got TB because of raw milk. And I know others who suffered from TB and yet they don't consume raw milk. If raw milk was causing TB then how come that there are those who suffer from TB and yet they don't take raw milk?

Respondent: Additionally, in regard to consumption of raw milk it is related to assisting in parturition without gloves. A lot of us agro pastoralists do not see any harm arising from raw milk and God is also helping us because as soon as our babies are weaned we give them raw each time they are hungry or fussy. And they continue taking raw milk until they are adults like us. And we don't notice any negative effects arising from raw milk consumption.

Interviewer: So if people here experienced some negative effects they would boil milk?

Respondent: Yes, we would boil the milk if that was the case. It's the same as when we are advised that malaria is caused by mosquitoes and to protect ourselves we need to use mosquito nets and we do use mosquito nets because we have suffered from malaria in the past many times. We are advised that if we use mosquito nets then we will not suffer from malaria although we still do. So all of us use mosquito nets. Malaria is still there because the mosquitoes here are stubborn.

Respondent: Also the herdsmen do not use nets and they hardly ever suffer from malaria and we here at home use nets and we still get malaria.

Respondent: With malaria we are well informed and we take malaria patients to hospital immediately unlike in the past where we wouldn't seek prompt care. And we would take them to hospital when they are seriously ill.

Interviewer: Talked to them about extra pulmonary TB and TB.

Interviewer: What would be the best way to educate local people so that they can begin changing their habits?

Respondent: I think that researchers need to identify which communities are afflicted by zoonotic diseases through e.g. consumption of raw milk and thereafter provide training through community engagement workshops. This education through engagement workshops would provide us with a chance to ask questions and thus gain more in depth information. Because we will be able to ask questions and get more enlightenment and maybe in that case we will change our habits and especially encourage the younger people; our children to boil milk before consumption. And this will go on with following generations.

Interviewer: What do you think about the use of radio to pass this information?

Respondent: Some people do not own radios. Others own them but they do not listen to them because they are busy tending to their flock. But with community workshops even if a man is unable to attend he could send someone else from the family to represent him and thus he will still get information on what was being spoken about.

Respondent: On the other hand, researchers need to identify whether zoonotic diseases are a problem in all of Tanzania among agro pastoralists or only in certain areas. But people definitely need more education.

Q4 Practices on herding and attitudes of herdsmen

Interviewer: During what months are cattle taken to the grazing area?

Respondent: From June until March of the following area or when flooding starts which can be even in April.

Interviewer: At what age do the boys start going to the grazing area to live there?

Respondent: From 10 to 20 or 30 years even. They usually go both younger and older men. They only come home to get food and treatment if need be. They mainly take milk alongside ugali or rice and fish too because there are fishermen in that area too.

Respondent: There is a way that they prepare milk too to eat alongside ugali. They boil the milk with some flour in it. It becomes a kind of porridge. They also cook fermented milk and take alongside the ugali.

Interviewer: How would you describe the health of the young men who go to the grazing area?

Respondent: They are usually in good health. They don't even get as sick as the people at home. They take dirty water for example and we can't because we get sick. They live far from each other so they don't pass diseases to each other too. The herdsmen hardly get sick.

Respondent: Like I said, in the past many young men were not going to school but now they do at least until class seven. Usually if they do not perform well then they go and continue to look after livestock but if they pass well then we take them to high school. If we don't have young men in our homes to look after livestock, we hire a herdsman and pay them each year. So there are not many 10 year olds who go to look after livestock in the grazing area. They only go there to take food on Saturdays to the herdsmen. They only look after livestock from 14 or 15 years old if they don't perform well in primary school. If we don't take children to school, the Government enforces it by arresting the parents so we have to enroll them on school. And if a child performs well in primary school and we don't take them to high school the government also arrests the parents.

Q5 Livestock Wildlife Interaction

Interviewer: Do livestock come into contact with wild animals in the grazing area?

Respondent: There are no wild animals in the areas surrounding the grazing land for this village. The Selous game reserve is far and ILUMA has no wild animals only grass. ILUMA came about during the land use management workshops and its related to conserving the Kilombero river. So that's how ILUMA came about to protect the river and separate the livestock and people from the river. We are not allowed to take our livestock to ILUMA. The only wildlife are crocodiles.

Interviewer: How are livestock transported from one place to another around?

Respondent: Cattle are walked through the roads and not in the bush so they don't come into contact with wild animals.

Q6 Common livestock diseases in this area and practices related to abortions in cattle

Interviewer: What are the common livestock diseases in this area?

Respondent: (Pause). Mainly FMD (Lusoona). This is the most common disease and we think it has no cure.

Respondent: We have asked livestock officers and they tell us this disease has no cure. We try to treat it in vain. It's a bad disease and occurs when there is flooding.

Respondent: Also CBPP where the cattle have difficulty breathing and coughing. This one we treat.

Respondent: There is also "ndui" and it causes abortions or calves that are born with body sores.

Respondent: There is also "Ndorobo" where cattle become emaciated, abort or give birth to weak and very small pre term calves. The cattle also have diarrhea. "Mbung'o are bigger than "Ndorobo". They are different. The former does not lead to adverse effects like the latter does. "Ndorobo" is more harmful and as soon as a cow is bitten it develops a rough hair coat. "Ndorobo" also lay their eggs on the grass and once cattle eat the grass then they begin to have abortions. We are able to differentiate between abortions caused by "ndui" and those caused by "Ndorobo". In the former the aborted calf and placenta usually has body sores but the latter the calf will be so small and the cow will be emaciated. On the other hand, the placenta may not come out too if the cow is suffering from "ndorobo".

Interviewer: Are there times when cattle abort and they don't have body sores or are emaciated?

Respondent: Yes because of "Ndorobo". Some cattle do not become emaciated but they still will be affected by the disease.

Respondent: Also in the case of CBPP, cows also abort if the cow is very sick. But with "Ndorobo" we know its because of "Ndorobo".

Interviewer: How do you dispose of still born calves?

Respondent: We burn them.

Respondent: We give them to the dogs. The young men just chop it up and give it to the dogs. Its cut up so that you share it out to all the dogs because we often own more than five or six dogs.

Respondent: Another reason why we cut it up is to prevent dogs from catching a healthy calf and eating it if we make it a habit to feed them a whole calf.

Interviewer: Is there any other disease that causes abortions in cattle, still births?

Respondent: Only that and “ndui”.

Q7 Perceptions on infertility in cattle

Interviewer: What do you think causes cows to be infertile?

Respondent: (Pause). There are infertile cows of two kinds. One kind (tasa) is the cows that have given birth two or three times in the past and then they stopped. Because they are mounted by bulls all the time. The other kind (mgumba) has never given birth because they lack a vagina. And the former can get very big and fat.

Respondent: Most of the time we sell them because they are very big and thus fetch a good price. The butchers like them because they are fat and big.

Q8 Perceptions on reduced milk production

Interviewer: What causes a cow to have reduced milk production?

Respondent: Its because of “Ndorobo”

Q12 Common human diseases in this locality

Interviewer: What are the common human diseases here?

Respondent: Malaria

Respondent: Another one is UTI

Respondent: Typhoid

Respondent: Flu

Respondent: TB also especially at Kibaoni and St Francis.

Respondent: Also lets not forget HIV/AIDs

Q 13 Signs of a febrile illness

Interviewer: What are the signs of a febrile illness?

Respondent: Feeling very cold, tiredness, fever, headache, stomachache

Q14 Treatment pathways related to febrile illness

Interviewer: What is the first cause of action when you have any of these signs?

Respondent: The first thing is to go to the health facility.

Respondent: The first thing is I take Panadol and if I don't feel better then I go to the health facility.

Respondent: Yes, most of us take Panadol, some get some herbs boil and drink and if still not well then go to the hospital. It all depends on someone's understanding. (Laughter). So we start either with herbs or Panadol.

Interviewer: Why do people use herbal medicine?

Respondent: Its like first aid.

Respondent: It also depends on ones' faith that if I use a certain herb I will feel better.

Respondent: Like when I was growing up our parents used aloe vera to treat diseases when we got ill. We had aloe vera in our farm. So even I if I had access to aloe vera I wouldn't use Panadol. Aloe Vera would be cooked and stored in jars.

Interviewer: Do you still use aloe vera?

Respondent: Aloe vera works well even for stomach issues but unfortunately we don't have it here in this area.

Interviewer: Do women also use herbal medicines?

Respondent: Yes they do. They also know these herbal medicines since they too understand them.

Interviewer: Do people here buy over the counter medicine without a doctors prescription?

Respondent: No the doctor has to do tests first so that he can issue the right medication. They have all the tests in the health facility.

Respondent: The only test they do is malaria. If they realize you have no malaria they give you Panadol only. But if you don't feel better and after like three days you go back to the health center and they test again you are told that you are suffering from malaria.

Respondent: Its only very rarely that they check for malaria and say you don't have it. Often they claim that you have malaria. And if you don't have malaria they send you to Kibaoni.

Respondent: Yes, if you take your child to the hospital they will claim that the child is suffering from malaria. And then you go buy malaria medication.

Interviewer: Where are the tests for the other diseases conducted from?

Respondent: Other tests are conducted in private lab facilities like in Lungongole there is one.

Respondent: Others go to Kibaoni since these private labs open and close and thus are not reliable.

Respondent: Most of those who go to Kibaoni are those who are very sick but if not they don't. Those who go to Kibaoni are very sick. Then there are those who always need the tests done every time they get sick and others do not. Very few like 30% go to Kibaoni.

Respondent: It really depends on what one is suffering from. Because what we have here in the village Is only a dispensary. So people go to Kibaoni.

Respondent: If anyone from the family doesn't get better after visiting this dispensary we go to Kibaoni.

Respondent: The clinicians in Sagamaganga dispensary usually advice us to either go to the lab or to Kibaoni.

Interviewer: What are the challenges you face after being referred to Kibaoni?

Respondent: (Exclaims) The queues are very long and it can take one a whole day without getting treatment or even two days. The only ones who are treated without delay are the pregnant women.

Interviewer: Do people but malaria medicine over the counter?

Respondent: It happens as a precaution when one feels the symptoms and the hospital is far.

Respondent: We would do that some years back when we didn't have the dispensary at Sagamaganga. Most of us don't do that anymore. In the past some people bought malaria medicines and stored them in their houses.

Interviewer: What happens when the young men in the grazing area fall sick? Where do they seek medical care?

Respondent: Like we said that doesn't happen often but when it does they come home and we take them to the hospital for treatment.

Interviewer: Do children also use herbal medicines?

Respondent: Herbal medicines were used in the olden days not now.

Respondent: Yes, even for children. There is a disease caused "degedege" which afflicts children and we use herbal medicine and they recover.

Respondent: Sometimes also if women do not conceive they use herbal medicines and they are able to conceive. And the herbal medicines help, so herbal medicines are used. Sometimes also if a woman is due to give birth and delays to its because she slept with more than one man like five men during that particular pregnancy. So without herbal medicine she cannot deliver. She has to name all those men and then she can deliver the baby. The older women tell her to name all the men she slept with and then she can deliver.

Respondent: Those are things that were done in the past not nowadays.

Respondent: These things still happen even now. They are real. Sometimes you find that a mother gives birth via a caesarian section during the first pregnancy then she goes to a herbalist and in the second pregnancy she delivers the baby normally. How can you explain that? Women are delivered via a CS because she had intercourse with several men when pregnant.

Respondent: These are things we have experienced and we know they are true. Its better to take your wife to a herbalist than for her to go to have a CS.

Interviewer: What are the challenges you face in accessing health care? Other than long queues

Respondent: We have to look for money to get medication and also drugs are readily available.

FGD Men Lungongole 6th Aug 2019

Question 1 Gender roles in livestock management

Interviewer: Why do you keep livestock?

Respondent: Livestock help us in the farm (cattle) and for livelihood. Its like a bank to us. It helps us to meet our daily needs.

Interviewer: Why do you keep cattle, goats and sheep and not one or the other?

Respondent: This is because problems are of various kinds, there are minor financial problems which one resolves by selling a chicken., if bigger you sell goat or sheep and major ones by selling a cow. We acquire new stock by buying more in the livestock markets. Those come from within the District. Not from outside the district.

Respondent: No, sometimes they actually do come from outside the district like from Mahenge in Ulanga and on to Kilombero.

Interviewer: How are the cattle transported from those areas to Kilombero?

Respondent: It depends on the number of cattle. If a few they are transported on foot if many they are transported via a lorry. This usually happens as people put their cattle together and transport them by lorry. The lorry is not allowed on foot at the Magufuli bridge so they have to be on the lorry to cross over to Kilombero. Cattle are not allowed on foot across the bridge. They say the cattle are damaging the bridge.

Question 2 Practices on assisting with parturition

Interviewer: Do you use gloves when you assist cows with parturition?

Respondent: We don't use gloves. We just use bare hands. Livestock officers tell us we should but we are used to assisting livestock without the gloves.

Respondent: I have never seen anyone use gloves, only the livestock officers use them. If I try to assist a cow and am unable to and I call the officers, they come with the gloves. We don't receive any gloves.

Respondent: They are sold in the shops but we are not taught to use them. We just use our bare hands and once done we wash our hands.

Respondent: You are the one who is telling us now.

Respondent: If you are in the wild and a cow needs assistance where are you to get the gloves(laughter). You just wash your hands without soap when done if you are in the wild.

Interviewer: Have you ever been taught the necessity of using the gloves?

Respondent: You see, here when the livestock officers call us for meetings most of the things they talk about is about diseases like FMD, CBPP, ECF especially if there are outbreaks but teaching about using gloves during assisted parturition that one doesn't happen. I have never heard it. You are the one teaching us now.

Question 3 Practices on residing with livestock

Respondent: Most of us leave far from the township so for us Wasukuma we build a big house and in it we sleep with livestock like calves or goats/sheep. The people sleep in the bedroom and the livestock in the open area. But now people are changing. That is very common among us pastoralists. Or you find that herds boys build a shelter in the cow shed and sleep there surrounded by cattle. So the person is in the midst of cattle all night. This is done for protection because in the grazing area there are a lot of dangerous wild animals. And so if a wild animal comes into the cow shed the cows will stir and the herds boy will wake up. So it is for safety that they do that.

Respondent: They just build a little shed to avoid being trampled on but they are in the midst of the cattle.

Respondent: This is our culture. The older men tell us that when you stay close to your livestock they know you and they like you but if you are a visitor they can trample over you because they don't know you. When as an owner you get in they come near you and receive you well.

Question 4 Perceptions on raw milk consumption, boiling milk and community engagement

Respondent: Tell us what about fermented milk, is it safe to drink now that we don't boil before fermenting it?

Interviewer: Its not safe to drink it raw and unfermented.

Respondent: Ah, But, this is very difficult for us to do. There are those who say that if you boil milk while you have calves the calves do not thrive. There is a tribe called "Mang'ati" and they claim that a calf does not allow you to boil its mothers milk. That is what we believe. We like to drink raw milk especially while its still warm right from the cow's udder. Livestock keepers claim that this milk is very nutritious and very tasty. However, during the RVF outbreak when they told us to boil milk and not eat meat we did because people were dying but once the RVF outbreak ceased we resumed taking raw milk.

Respondent: And to us boiling milk then was seen to be such a bother.

Respondent: And you know once you boil milk and remove the cream you have removed all the essential nutrients so it is like drinking water. That is why we drink raw milk. Raw milk also destroys all the harmful germs and toxins in the body too. Yes, indeed because when you consume any poison you quickly look for raw milk and consume because it destroys the poison. Have you never heard of that? Now when you boil the milk you destroy the milks ability to neutralize the toxins.

Respondent: Something else is that women need to be educated about the need to boil milk too because they are the ones that play the biggest role in this. As far as boiling milk is concerned. The are the ones feeding raw milk to the children.

Interviewer: However, men are the decision makers…

Respondent: That's true but even if I am sensitized and my wife is not and I go and tell her that she needs to be boiling milk and she herself hasn't been taught what do you expect will happen? She will say that the man is harassing her with unnecessary demands. Let me tell you, some women are interesting because they only take milk from indigenous cattle and not modern cattle. They need to be educated especially for the sake of the children because women are the ones that stay home with the children. So if women are educated and they understand the dangers of raw milk use and they change then it is easy for the men to follow suit. But educating the men only…. men are not easy to be convinced.

Respondent: This researcher knows our culture that we as men are the decision makers in our homes and we play a big role in what is done or not done in our homes. We will question why our wives are changing and ask them who should make decisions at home.

Respondent: That's true but am asking that we both be educated because as men we are not always at home. We are here in the shopping center and women are at home and can boil milk for the children.

Respondent: What if you leave instructions at your home that she must boil milk?

Respondent: Yes, you can do that but then your instructions can also be ignored.

Respondent: This education is needed for both men and women.

Interviewer: I think also that men need to be very included in this process because men are the main decision makers and stakeholders and if you institute it then it will happen. Men as the leaders of the households have a big role to play.

Respondent: Yes, that's true and we let our wives go for example for post natal care and we don't prevent them. The women need to be educated too. Because it Is true as you are saying that some people get ill and they take a long time to recover and we don't know what they are suffering from. And it could be diseases like what you are saying, brucellosis for example. I have a question too; some people are advised in the health facilities to consume fresh boiled milk to treat stomach ulcers. As we know even unpasteurized milk is fresh milk, can that also not cure those stomach ulcers?

Interviewer: Fresh milk means the boiled milk right?

Respondent: Yes, the boiled milk but you find some people also call raw unfermented milk, fresh too. So I don't understand because they are told to use fresh milk while I know that even raw unfermented milk has no harm at all. So I wonder why the distinction.

Respondent: As she said the raw milk has bacteria so it is harmful. It has bacteria.

Interviewer: Do young people especially the herds boys know about the need to boil milk?

Respondent: Ah, those ones have absolutely no idea! Those ones cannot boil. They can milk and take two liters of milk immediately to quench thirst. They mainly consume ugali with raw milk. Or sometimes they cook milk mixed with a little flour and then that becomes like a kind of porridge. But most of them consume raw milk.
They do not have clean sources of water. They drink water from the same dams where cows are watered and they dig shallow wells so milk is used as water to quench thirst.
Interviewer: You say that you have been educated on the importance of boiling milk to reduce the risk of disease. Please tell me more about why you still do not boil milk?

Respondent: You know it is just our practice. Because for example they tell us that we should not feed raw milk to children because children can get sick quickly but in reality for us livestock keepers we milk our cows and the children are nearby. So we milk and immediately serve the milk into a cup for the children and they drink it. (Laughter). They do not even sieve it.

However, the biggest contributor is lack of formal education. 95% of us are not educated so maybe the future generations will boil. This means that because people are not educated they do not understand the things they are taught.

Respondent: Because of low education people do not understand.

Respondent: Now you see, unless I insist forcefully in my household…….. because you can educate someone not to smoke but they will tell you “I have smoked for many years and I am not ill”. Then when you ask them,” have you ever been tested for disease, how sure are you that you are not ill?” Then they will ask why should I get tested when I am not sick. So it is the same thing that people don’t take these things seriously. So maybe those who are the current generation and in the future because they are going up to class 7 or form 4 they will do things differently. These things need some expertise so if one never went to school you cannot understand and it becomes only a bother when one is told to boil milk. And all the time we are being educated to boil milk until…put it on a fire until it boils completely and the cream rises that’s when all the germs in the milk die then cool it for the baby or child to drink. But now when you advice someone to do that they tell you that the cream that comes on top after boiling is what is nutritious and so if you remove it then you are losing essential nutrients (laughter). So they claim that if you feed the baby the boiled milk in which the cream has been removed it’s the same as feeding water to the child.

Interviewer: What are some of the ways you propose would be more effective to educate people about these kind of things?

Respondent: You see we are being educated all the time by experts from Universities and other institutions but changing behavior is very hard. Unless we are taught every day not once in a while. Maybe in that case we can change. It’s the same as what we do with growing rice. People are being taught to plant rice using seedlings and not by scattering the grain. However some still use the scattering method and ask you, ”since I was young this is the method we have used and it has worked why should I change?”. They say it’s a waste of time, changing behavior is very hard.

Respondent: Unless we were examined and then we were told for sure that we are ill as a result of raw milk consumption. In that case we will change our behavior very fast. In that case we would be convinced but just telling us we are not convinced.

Respondent: Yes, if that happened and some were found to be sick from raw milk consumption we would indeed start to boil milk. We would change immediately. We would learn and start to boil milk. So not just being told but confirmation.

Respondent: You know when experts examine you and find that you are suffering from TB of the lungs or in the skin the tests do not show that you are sick as a result of raw milk so they examine as they do their research they know that diseases are a result of raw milk. So we wont still be convinced that the disease is a result of raw milk we will just say that these are diseases like all the others.

Respondent: But the way I understand it when researchers do their work they know what causes disease. Like in the case of malaria they know it is caused by mosquitoes. So you can't get examined and told you have malaria and they don't know what caused it. So in that same way they can tell what diseases are caused by consuming raw milk.

Interviewer: Have you ever heard of diseases that can be transmitted from livestock to humans?

Respondent: Yes, we have but scantily. There was one year in the past they were telling us there was a disease called…(pause) Rift valley fever. They even told us not to eat meat. That is one of the diseases. But we had never heard of it before, we only got to know of it that time. Then there is a disease in milk that they call…(pause)…it causes chest problems in humans…I think its TB…but we don't know since we are not experts. But they say that, the experts themselves. They tell us to boil milk and we are not used to boiling milk. We don't boil milk. We don't boil milk but we are used to not boiling. Also we have not come across anyone who has had a disease because of consuming raw milk so we don't boil it. We find it a bother to boil milk and have to wait for it to cool down before drinking the milk. Its just unnecessary trouble. You see…..

Respondent: We just sieve the milk to remove the dirt and cow hairs that get into the milk as you milk. But we don't boil, its not in our practice to do so.

Question 5 Practices on herding and attitudes of herdsmen

Interviewer: At what age do boys start going to live in the camps and look after cattle?

Respondent: Usually from age 15 onwards up to 30 years old. They go to the camps from June to April of the following year.

Interviewer: What kind of shelters do they build to live in?

Respondent: Those are the ones we were telling you live amidst the cattle. They sleep inside the cow shed.

Question 11 Livestock wildlife interaction

Interviewer: Are there wild animals in or close to the grazing area?

Respondent: Yes, they are there like buffaloes, ("sheshe") puku, lions, hyenas, warthogs and wild pigs. They mix and graze in the same areas.

Interviewer: Do you think there is any harm in livestock and wild animals interacting that closely?

Respondent: We don't know because we see such issues like cows aborting so we don't know whether it is caused by this interaction or by "Ndorobo". We don't understand. But harm is there. You can find wild animals that are ill and unhealthy in appearance and so we think they might infect livestock too with those diseases.

Interviewer: Are the game areas and livestock areas delineated?

Respondent: Yes, they are but the wild animals encroach into the livestock grazing areas. They come especially at night and they interact together. We are only wary of lions because they attack our livestock especially at night but ("sheshe") puku they don't cause any harm.

Interviewer: Farmers/ livestock keeper's spaces

Respondent: Farmers have encroached into our grazing area illegally thus reducing the available space for livestock grazing. Kilombero river is close to the grazing area like 1- 2km away.

Interviewer: Please tell me more about the interaction between livestock and wildlife

Respondent: In ILUMA there are buffaloes, ("sheshe") puku, wild pigs, warthogs, lions, hyenas and elephants too though these are rare because they would need to cross Kilombero river first. Livestock are not allowed into ILUMA and they hardly ever get in there. Livestock from this village do not but those from Ulanga do go into ILUMA especially at this time of the year. There is no drought but the grazing area in Ulanga is limited because the SELOUS game reserve covers a huge area so during the dry season in Aug-Sep they come into ILUMA. They do it illegally and then they disappear and then we are accused because they come close to our grazing area. There are also fishermen in Kilombero river. There is interaction between wild and domestic animals because there is no fence delineating the two areas. And they consume water from the same dams and rivers. Wild animals come at night to take water. There is a river separating our grazing area and ILUMA and its called "Matete." Water is a lot and we don't have a problem in that area. The grazing area is not sufficient…

Respondent: It is sufficient only that other livestock keepers bring their livestock here too. Livestock here are slightly more than 1000 and the grazing area is about 12,000ha but it has been reduced to like 3,000ha or 7,000ha. This was not done legally though. The grazing area is about 3-5 km from here. Cattle are in this area from March to June and they are here only for two months. The herds boys go there all year round unless they are married then they are allowed to come home often. Some go and stay there continuously for even 3 or 5 years. This only happens if there are no floods. When they come home they graze near the homes because they are not allowed into the Nyangaje forest. And this forest is heavily guarded.

Respondent: In the past we had a lot of livestock and thus a lot of milk. These days the cows have reduced. We have grown up with lots and lots of cattle. We didn't even use to farm because our fathers could sell cattle and buy all the basic needs. When I got married in 1987 my father sold a cow and he bought a bed, mattress and a new bicycle. And he needed us to maintain proper hygiene. And during the drought season he would slaughter cows and we would have enough meat. Even now we slaughter for our relatives and family members when they visit. We slaughter a cow that is not too old because the meat is tender. Those days we would slaughter a cow when a new baby was born and the new mother would have soup made for her and they would do this for each mother even with our big families. Cattle now are not many. Its not like in the past. These days most of our children are going to school so things are changing.

Respondent: Livestock officers do not visit us. They only come here if they want to apprehend cattle that have encroached into game areas or the forest because they will get paid. They do not come to educate us. They only come when there are opportunities for them to make money.

Question 7 Common livestock diseases and practices related to abortions in cattle

Interviewer: What are the common livestock diseases in this area?

Respondent: We treat most of the diseases ourselves but the most common is FMD especially during the heavy rains in March and April. We don't understand this one and it really bothers us. We treat it but the treatment is uncertain, its more like a prevention. Even that is not so good. That is the biggest challenge. Also cows give birth to still born calves and they also do abort.

Interviewer: Where do you buy the drugs?
Respondent: We buy from local shops around. They guide us on how to use the drugs but we also are like "doctors" because we have grown up in this environment and have done it for a long time and we advice each other.
Respondent: Usually when they abort they do not show any signs of disease. They will be appearing healthy and strong and then they abort. They appear healthy but then they abort. Most times we do not understand…because we usually have treated them. There are insects that are found in the wild and we call them "Ndorobo"..so we notice that we have already treated them against this disease at the right time but they still abort. So we don't know what happens. We don't understand because we have treated them and the abortions still happen and we have treated them. When a cow aborts we treat it again for "Ndorobo" or we use other medicines like "bero".. you see. And once we treat they get well and conceive again.

Interviewer: Please tell me more about abortions is cattle?

Respondent: And when we go to the medicine sellers they tell us that "this is the right drug to use to prevent abortions" and we inject the cows but the results are still the same. The cows abort. No change, you need to help us. Abortions happen all the time.

Respondent: But usually in July when the pregnancies are mid way like 5 or 6 months gestation. Abortions happen a lot during this period.

Respondent: I think the one where cows abort is quite different because when they abort they do so at about 4 to 5 months' gestation or even at 6 months. This is another disease and I don't know it for sure. We just say that my cow has aborted.

Question 16 Perceptions on infertility in livestock

Interviewer: What causes infertility in livestock?

Respondent: As we know it, most of these cows are too fat and so because of the fat they cannot conceive and they also mate repeatedly with the bulls. So we use them as draught animals and then they lose weight and conceive. We do that if we love that particular cow and do not want to dispose it. Often after a few months the cow conceives and gives birth.

Respondent: Sometimes you love that particular cow because it produces a lot of milk when it gives birth so you don't want to sell it.

Interviewer: That means the cow had given birth before…

Respondent: Yes, and you have already identified that it produces a lot of milk and it is a calm cow.

Respondent: You know some cows you want to have them because they are attractive and big and are good leaders. The cows that we sell easily are the ones that are troublesome and unruly. Those we slaughter easily or sell them.

Interviewer: What happens after you've used the cow as a draught animal and it still doesn't conceive after a few months?

Respondent: At that point you sell the cow or slaughter it when you have an important function at home. If you have an important visitor that you haven't seen in years like a parent or sibling, you slaughter the infertile cow. Having an infertile cow is a big loss because it is not reproducing and you cannot use it as a draught animal for longer than a few months or a year. Its health deteriorates fast. Because that is hard work and if a cow is already pregnant they suffer an abortion because of the hard work.

Question 9 Perceptions on reduced milk production

I: What causes reduced milk production in cattle?

R: This could be a result of worms' infestation which you treat. Also worms cause the cow and calf to be weak and they recover.

Question 10 Perceptions on hygromas

Interviewer: Have you ever witnessed cattle that have swellings on their joints? What causes this condition?

Respondent: Yes, we have. It is not common here though, its very rare. It is mainly in Pwani region. We don't know what causes it. We see very few cases here. I once saw a like three cows in one household that had joint problems. But its rare and we wondered what the issue was. Maybe it is a specific breed.

Question 11 Perceptions on retained placenta

Interviewer: What causes retained placenta?

Respondent: We use drugs and inject and if it doesn't work we use a stick and wrap it around the placenta hanging at the back and pull it gently over a few hours and eventually it comes out.

Question 12 Perceptions on the birth of a weak or still born calf

Interviewer: What causes weak calves or still born calves?

Respondent: Most of these cases are caused by "ndorobo" especially if the cow has been bitten by those insects and you did not treat it in time. Or also when you milk a heavily pregnant cow a weak calf is born but a still born calf is because of "Ndorobo". There are also cases where the cow is in a long hard labor or the cow is ill too. But I don't know what disease in particular because there are many diseases but most say it is because of Ndorobo.

Question 13 Common human diseases in this area

Interviewer: What are the common human diseases in this locality?

Respondent: Malaria and UTIs and typhoid.

Question 14 Signs of febrile illness

Interviewer: What are the signs of febrile illness (homa)?

Respondent: Fever, weakness, fatigue, joint pains. The first signs can be flu and tiredness.

Question 15 Treatment pathways during febrile illness

Interviewer: What is the first course of action you take when you have these symptoms?

Respondent: The first thing I do is go and get tested for malaria in a lab around. If no malaria, we test for UTI. If none then we get tested for typhoid.

Respondent: If all three are not found they advice you to go and use herbal medicines.

Respondent: They give you medicine and tell you if you don't feel better you go to a bigger facility and do further tests. They give you medicine first and treat you according to the symptoms first. There is a private lab facility here and the closest public facility is in Sagamaganga. You have to go to Kibaoni. In Kibaoni they test for diabetes, anemia and conduct tests on the stool too.

Interviewer: Do people here purchase over the counter drugs with no tests conducted or before visiting a clinician?

Respondent: Yes, because from the symptoms one can conclude that they are suffering from malaria. Malaria makes one very tired, have a fever, vomit and have diarrhea.

Respondent: Once you suspect its malaria and go to a health facility they tell you you are suffering from malaria so next time you have similar symptoms you conclude its malaria you are suffering from.

Respondent: No you can't do that because malaria and UTI have similar symptoms. I had such symptoms recently of fatigue and fever and when I went to the hospital they told me I was suffering from UTI so if I had bought over the counter drugs for malaria I would still be unwell.

Respondent: However, clinicians give you both malaria and UTI drugs so that if you don't have malaria then the UTI drugs treat you.

Respondent: There is UTI that is severe and causes kidney failure. Doctors have to test you properly therefore to determine what you are suffering from.

Respondent: UTI is so common that almost all the time you are diagnosed with that disease. It is very common these days and you have to get the right medication including injections. If you are treated for malaria and non severe UTI and still ill then it means that the UTI you are suffering from is severe and you need better treatment.

Interviewer: Are you able to tell that you are suffering from UTI without going to see a clinician?

Respondent: Yes, you can because with UTI you urinate frequently and there is pain as you finish urinating. Then you know it is UTI.

Respondent: With UTI you need to go to the bathroom to urinate up to 4 or 6 times per night then you can tell you are suffering from UTI.

Interviewer: What about typhoid? Can you tell?

Respondent: You can but with typhoid its trickier because you feel the symptoms of a febrile illness and maybe buy malaria drugs and not get better. And you continue to get worse so in that case you go to the hospital and get tested.

Interviewer: At what point then do people generally go to the hospital?

Respondent: It is not often that people here go to the hospital as the first course of action. Usually people buy malaria drugs or “metacelvin” and if they recover fine if not after a week they go to the hospital and often they are told its UTI they are suffering from.

Interviewer: Do people here use herbal drugs to treat febrile illnesses?

Respondent: Yes, like “mwarobaine” is very helpful. You boil and drink and use it to treat malaria. You can combine this with “mpera”, “mkwaju” and another one called “mbingombingo” called “magugu” in Swahili. You combine all four and they are very helpful for malaria.

Interviewer: Are these herbal medicines used for children and women too?

Respondent: For women yes but for children (pause)…its very bitter…

Respondent: It depends actually because if one lives far they have to use it even for children ages 5 and above though.

Interviewer: Do you ever use herbal medicine alongside conventional medicine?

Respondent: It depends on someone's faith. Some people have a lot of faith in herbal medicine so they have to use them alongside the hospital medicine. For some people they don't think they will get well if they don't use herbal medicines so they combine them. And then those who prefer hospital medicine believe that these will make them get better. So some combine and some take either. However, for us livestock keepers we like to use herbal medicine and we consider it like Panadol of sorts. When you get ill you go get a herb from the forest and boil and then take it. We know that herbal medicines work well and so we use them. They cure diseases. For example, if one gets a snake bite even from a very poisonous snake they take herbal medicine and they recover completely. If you go to hospital because of a snake bite you could have a limb amputated but with herbal medicine you recover completely. If one has the medicine for snake bites you take it immediately after getting bitten and if not, you make small lacerations close to where you were bitten and in that case the venom does not spread throughout the body. The medicine you take prevents the venom from spreading and the herbal treatment you use on the lacerations prevents the spread. We get bitten all the time and keep the medicine at home. Another reason why we use this medicine is because the health facilities are far and transportation is a challenge too so if you wait to go to the hospital you definitely will die because the venom is very poisonous. So you must have the herbal medicine nearby and use it in case of snake bites for both humans and livestock. We carry it around and make sure at least five people in the village have the treatment at any given time. We like the herbal medicine because they really do work. Its only for some diseases like HIV that they don't work but for other small diseases like gonorrhea….. those are small diseases that are treated with herbal medicines. Also there are snake bites that are unusual and these are caused through witchcraft when your enemy sends the snake to bite you. This kind of snake bite is very hard to treat and you can die unless you get a specific traditional healer who specializes in that. And in that case the medicines do not work including the herbal medicine. So herbal medicines really help us. About 80% of the time they do work and treat disease like I told you. Even in issues of infertility in humans or when you want to get a baby of a specific gender we use herbal medicines.

Interviewer: Do people visit traditional doctors when they are ill?

Respondent: Yes, they do.

Respondent: A traditional healer and a doctor are very similar. The only difference is that the doctor can perform certain tests that a traditional healer cannot. But the traditional doctor also is knowledgeable based on experience because they see a lot of patients.

Interviewer: It appears to me that you utilize different methods of health care from over the counter drugs to traditional healers and clinicians…

Respondent: Going to the hospital…we only do it once a disease becomes very hard to treat and after you have exhausted all the options. And also once you test for all the diseases even at the hospital like HIV, Diabetes and you are not getting better then you go to the traditional healer for treatment. And people get better.

Question 22 Experiences with formal and informal healthcare

Interviewer: How easy is it to access formal healthcare?

Respondent: It is not easy for sure because we livestock keepers often live far from the facilities. So when one goes to the grazing area we carry medicines for stomach illnesses, snake bite medicine and others too…even panadol and malaria drugs. You store them in your bag and use them as needed. Other diseases you just dig up or gather more herbs. One has to do this because when it floods its difficult to access transportation and so having those medicines is very helpful. Transportation is a challenge during the rainy season.

I: What is your experience of hospital services in this area?

R: Its okay…but some people do not like the experience of getting poked and injected which they do in the hospital so they prefer the herbal medicine.

R2: For example, for me I remember it was in 1987 when I last got an injection. I have never been ill enough to need that. Some people do not like that hospital experience at all.

R: I went recently to the hospital to have a tooth extracted and due to my experience I decided I will never go to the hospital again. The anesthesia didn't seem to work and it was very painful and the clinician was not at all gentle. It was very painful and I really wondered why I took the trouble to go to the hospital and possibly die there. And yet there are sufficient herbal medicines I could have used. I decided that I will never go back there again. Not for tooth ache, never again. Someone pokes your mouth until your mouth is numb and he was not gentle. I think that he was a student.

R3: Probably they should have used a lot more anesthesia.

R: It was a private clinic and it was not a good experience as described by my friends it would be. I have really thought about it and there is nothing great he did other than the anesthesia, that is something a traditional healer could easily have done. It was a bothersome experience then I was given amoxyl and Panadol and I have recovered now. If you find a trainee it is very hard. You get a trainee who keeps poking you to find the right vein over and over…he pokes you severally and it is so much trouble…it's a bother. You can't have faith in them. We only go to the hospital if you have no other choice. People die during operations too because these clinicians are inexperienced. A boy recently died while undergoing a hernia operation. And the boy died out of the clinicians' carelessness. You know they are inexperienced and are given treating responsibilities right after college. You know head knowledge is very different from actual practice. These are the kinds of things that make us not trust the formal healthcare system.

R4: The only solution is for the family of the patients to bribe the experienced doctor to attend to their patient. If you don't do that your patient will die. You have to bribe them especially in the case of surgery. If you don't students are made to do the operation and you die. And to them these kinds of death are not a big deal. So we have no faith so they try other options first before going to the hospital. And you know the only hospitals here are Sagamaganga and Kikwawila dispensaries. It's a big challenge.

Sagamaganga 16/8/2019 FGD Herdsboys

Question 1 Gender roles in livestock management

Interviewer: Are you the ones who milk the cattle?

Respondent: Yes, we start milking at the age of 10.

Interviewer: Do you also treat cattle when they are sick?

Respondent: Yes, we start treating cattle when we get to 10 years of age.

Interviewer: Can you slaughter too?

Respondent: No, we cannot do that yet.

Question 2 Practices on assisting with parturition

Interviewer: You said that you can do a lot of livestock related duties from the age of 10, can you help with parturition at that age too?

Respondent: Yes, from the age of 13 years old we do.

Interviewer: Do you wear gloves when you are assisting with parturition?

Respondent: No, we do not. We use our bare hands

Interviewer: Do you think there is any harm in helping with parturition without wearing gloves?

Respondent: No, there isn't. That is all we know how to do (laughter)…to help cattle to deliver. There is no harm at all. Once you finish helping the cow to deliver you wash your hands and that is it. You are fine. You just wash your hands with soap.

Interviewer: Do you always wash your hands with soap?

Respondent: Yes, some of us do not wash hands with soap. Others here do not even wash their hands; they even go to bed having not washed their hands. They eat with those same unwashed hands. Actually when a cow delivers while in the grazing area we just wash hands in the streams and that is it and then go ahead and eat. We drink water using those same hands. Others cook without having washed their hands. (laughter).

Question 3 Practices on residing close to livestock

Interviewer: Do you ever sleep with or close to cattle?

Respondent: Yes, it happens. We do this when its too hot inside like in the month of Sep it is very hot inside the shelter. We also sleep close to the cattle to avoid animals like snakes which can bite us if we sleep further away from the house. And we also sleep with a torch nearby so as to identify any harmful animals and thus keep safe. We sleep close to the calves because it is safer. The cattle like to push and shove each other at night and if you are too close to them they can hurt you by trampling on you thus poking your eyes or breaking your bones. One of us here was trampled by a cow at night while he was sleeping close to cattle and he was also hurt in the neck so now he does not sleep close to cattle any more.

Respondent: Something else, here in the camps we do not like to shower. We can go up to one week or four months without taking a bath. And we find it ok to stay that long without a bath. But when we are back home and we take two days without taking a bath we fall ill. That really perplexes us. Here in the camps we shower only rarely. Here we stay dirty and we hardly ever fall sick. We can stay up to a year without taking a bath. At home it does not happen. Here we shower in the rivers or we just dip ourselves into the dam and walk out and claim to have taken a bath. Herdsmen do not shower often because even the cattle can hit you because they fail to recognize you. We hardly ever get sick here too. And when we feel ill we take Panadol. We always carry Panadol.

Question 4 Perceptions on raw milk consumption, boiling milk and community engagement

Interviewer: Do you boil milk or you drink it raw?

Respondent: No we do not boil milk, we drink it raw.

Interviewer: Do you know the importance of boiling milk?

Respondent: Yes, we do. Once you boil milk and drink it it is good because after you drink it, it goes and kills the bad organisms in the stomach. I heard this……i used to boil milk at home. I used to see others boiling milk too.

R2: No we do not. This one here is confused. We do not understand him. Milk that has been boiled does not kill any bad organisms. However, milk that has not been boiled is good because even if you have consumed anything poisonous the raw milk neutralizes the poison.

Interviewer: What kind of poison are you talking about?

Respondent: Even pepsi (soda)…. these drinks which have chemicals…actually anything that has chemicals in it. The raw milk helps to deal with the harmful chemicals so that they do not harm you.

R3: Boiled milk is good because it kills organisms like germs that cause diseases.

Respondent: I think that boiled milk is good for people who have stomach ulcers as it treats them. But…I think it depends on individuals if they like it boiled or not. If you boil milk, then we use it as tea. It's a matter of personal preference. However, generally we consume raw milk.

Respondent: We like raw milk.

Respondent: We just like it. Boiling milk is a lot of work. We consider it to be a lot of unnecessary extra work. Because you have to do it each day….. but if you have stomach ulcers boiled milk is good for you.

Respondent: I cannot boil milk because once milk has been boiled it looses taste. So I would rather take raw milk.

Respondent: Boiled milk is ok and tasty. I don't boil it but I don't mind it either.

Respondent: If you are used to boiling milk you will find it ok but if not you find it unappealing. It is just what one is used to. I personally do not like it at all.

Respondent: We mainly eat ugali and rice. Usually we consume both with milk. We only eat fish and beans very occasionally. Most of the time it is milk with ugali and milk with rice. We take fresh milk as well as fermented milk.

Question 5 Practices on herding and attitudes of herdsmen

Interviewer: What is the age group of those who come to herd cattle in this area?

Respondent: Between 13-19 and some up to 30 years of age.

Interviewer: Where do you graze your cattle?

Respondent: They graze in the farm areas after people have harvested. We water them in the rivers nearby. There are many rivers around here most of the year round. We graze where no farming is done.

Respondent: When it floods we take the livestock back home and graze them…. we just struggle looking for pasture in the places where there is no farming taking place. Usually we graze livestock within our own homes or in the areas between farms.

Respondent: We bring the cattle to this grazing area in June and July.

Respondent: We stay here until March or April of the following year. We leave when the flooding intensifies.

Respondent: We stay for the entire duration.

Respondent: And we come each year.

Respondent: We sleep in the small houses you see here. The cattle sleep outside in the open while the calves are sheltered in the holding pen just outside the house so that they do not suckle all night and we have no milk in the morning.

Respondent: We stay here for as long as possible, sometimes until the water gets to our shoulders. That is when we leave and go back to the village.

Respondent: Sometimes our families have to send people with dhows to rescue us. However, if one knows how to swim then it is easier to get back home.

Respondent: If the rains are not too heavy then we stay here the entire duration and we don't need to go back home. We have to go home at that time because even for the cattle it becomes impossible to graze during that period. And cattle are not able to feed when there is that much water on the ground.

Respondent: Yes, we do graze cattle along the river banks during the day while the hippos eat in the same area at night. The hippos only eat cow dung when they lack food.

Respondent: We do not water cattle in the bigger rivers because we are afraid of the crocodiles. We take the cattle to the dams. We only go to the rivers when there is drought in Sep-Nov. During that time the water in the dams gets less and dirty and so in that case we have to take the cattle to water in the rivers. We take them to river Maweni during that time. Cattle do not like to take dirty water. The hippos are in Kilombero river though and they come all the way to this place. Its about 4 km from here. We border the game reserve called ILUMA. Once you cross river Maweni then you get into ILUMA and after that you get to the Kilombero river. There is a shopping center in that area of Kilombero river called Nanjogolo where there are fishermen too.

Interviewer: Do you ever take your livestock during the flooding season to the Nyanganje forest?

Respondent: No, we never do. We are not allowed in there. If you are caught there you are apprehended and all your cattle sold.

Question 6 Livestock- wildlife interaction

Interviewer: Are there any wild animals in this locality?

Respondent: None in this area. They are only in the wildlife conservations area. There are none here. Only monkeys.

Respondent: No there are no monkeys here too. Only pythons, crocodiles and hippos especially when it floods. Hyenas and lions come here too when it floods. They come because there is no food available at that time. So they come to hunt the cattle. This happens when the game areas start flooding like the rest of this area.

Respondent: Yes, there are. Wild animals like hippos, hyenas and lions do come to our area. They sometimes kill and eat the cows but the hippos usually only come to feed on cow dung.

Respondent: Actually I have been chased by a hippo one time. However, hippos are not aggressive most of the time. They just come to eat cow dung. They are only aggressive if you attack them. They are very fierce too all the same. You know hippos feed on grass like cows so when there is flooding then they also lack feeding grounds and thus they come looking for cow dung. Sometimes the hippos go all the way to peoples farm looking for pasture. Once the flooding subsides then you cannot see any hippos here. Hippos feed on the grass that is along the river banks. And when it floods they go to the rice farms. Crocodiles and hippos are many here but they bother us only when it floods. Also crocodiles and hippos do not stay in the same dams because they are enemies.

Respondent: As they said we encounter crocodiles, hippos and lions. Most of the other wildlife are in the game reserve in Selous.

Question 7 Common livestock diseases and practices related to abortions in cattle

Interviewer: What are the livestock diseases that are common in this locality?

Respondent: Ndorobo which manifests as rough hair coat, animals become weak and CBPP whereby the cattle cough. Also eye infections, emaciation, worms, foot and mouth disease especially during the flooding season.

Respondent: There is another disease we call "mashioda" which refers to cattle having difficulty walking. They sort of limp(laughter). And also there is the problem of snake bites because our cattle often get bitten by snakes.

Respondent: Yes, that is a big problem. There are a lot of venomous snakes here.

Respondent: Usually this happens at night when the cattle go out to seek pasture at night. However, even during the day they get bitten by snake too. Snakes are so many here. You find them all of over the place. (Exclamation).

Respondent: Like me I have gotten bitten by a snake some time ago. I was treated by a traditional medicine man and I got well. There is a man who really knows how to treat snake bites. And we recover.

Respondent: Snakes here are dangerous; they can bite you repeatedly up to three times if you are not careful. They bite once then the fang breaks and another one grows immediately and on and on it goes. The fang gets into the blood stream once the snake bites you. If the snake bites you three times you cannot survive. They even get into our houses when it floods. So we have to use a torch at night to keep away from them.

Interviewer: Do you treat the livestock when they are sick?

Respondent: Yes, we treat them ourselves.

Interviewer: Why do cows abort in your opinion?

Respondent: It happens when you do not vaccinate them for Ndorobo and the abortion happens when the cow is in the advanced stages of pregnancy like 7 months onwards. The cow will not have become emaciated but will be appearing strong.

Respondent: (PAUSE) Only Ndorobo causes cattle to abort.

Respondent: After an abortion we bury the aborted fetus. Some give it to the dogs and others burn it. We cut it into pieces though before feeding it to the dogs.

Respondent: We cut it so as to drain most of the blood. Other times we cook the fetus before feeding it to the dogs. We cook it…so that we can also taste it (laugher). It is easier for the dogs to eat when it has been cooked. Additionally, the reason the cow has aborted might be because the cow was sick and so the fetus is infected too. So if the fetus is not cooked the dog can get the same infection that was in the cow.

Respondent: We are often the ones that cut up the aborted fetuses.

Respondent: No there aren't any dangers associated with handling aborted material. This is a common practice here.

Question 8 Perceptions on infertility in livestock

Interviewer: What about a cow that is infertile, what would be the cause?

Respondent(Pause)…However to us what we do is that we take the infertile animal to be a draught animal and after a while the cow conceives and gives birth. That is what we know to do.

Respondent: An infertile cow is because it is too fat. Too much excess fat is the cause so once you take it to farm then the cow looses weight and is able to conceive. This excess fat prevents conception from taking place.

Respondent: This can also happen due to the cow mating too often and repeatedly until it is unable to conceive.

Respondent: We sell infertile cattle to the butchers.

Question 9 Perceptions on reduced milk production

Interviewer: What is the reason for reduced milk production in a cow?

Respondent: This is because as the calf grows older the mothers milk also reduces.

Respondent: This happens when a farmer does not spray his cattle and so they are repeatedly bitten by insects like Ndorobo. But if you don’t neglect them then you will always have enough milk.

Respondent: Another reason could be inadequate feeding. In that case it often leads to reduced milk production.

Respondent: Also when a cow is pregnant there is reduced milk production.

Question 12 Perceptions on birth of a weak or still born calf

Interviewer: What is the cause of a cow giving birth to a weak or still born calf?

Respondent: This will be due to Ndorobo.

Question 13 Common human diseases in this area

Interviewer: What are the common human diseases in this locality?

Respondent: Malaria, stomach ulcers, fungus, worms, headache. Fungus is a big problem (“manyungunyungu”). This is because once you treat it on one spot then the infection spreads, often to the legs and sometimes also to the hands.

Q14 Signs of febrile illness

Respondent: The most common signs of ("homa") febrile illness are feeling cold, tiredness, reduced appetite, vomiting, staying out in the hot sun, feeling dizzy, headache, stomach rumbling.

Question 15 Treatment pathways during febrile illness

Interviewer: What is the first thing you do when you feel this way?

Respondent: We go to the hospital.

Respondent: Some do not go to the hospital. Once they feel these symptoms they just go to the shop and buy malaria drugs. They know it is malaria so they buy ("mseto") malaria drugs.

Interviewer: You are saying that you hardly ever get ill when you are here in the camps?

Respondent: Yes, we do not get ill, only very occasionally.

Interviewer: What happens when you get ill and you are here in the camp?

Respondent: We have mobile phones so we call home and they send a motorbike to pick us. They take us to the hospital where you get tested for malaria and treated. You take the medications and get back to the camp.

Interviewer: Do your relatives ever buy you medications without sending you for any test?

Respondent: Ah, yes all the time. Actually that is what they do most of the time.

Respondent: Once you take medicine and do not feel better then they come and take you to the hospital.

Interviewer: So you said the first thing you do is take Panadol, then malaria drugs and go to the hospital thereafter if you do not get well?

Respondent: Yes, that is what we do. Most often they just send us malaria drugs. Malaria is common here and though we use mosquito nets we still get malaria. And also we use nets that are torn.

Interviewer: What happens once you are taken to the hospital?

Respondent: They do some tests and then treat you.

Interviewer: Do you ever use herbal medicine to treat febrile illnesses?

Respondent: Yes, we do. We get the herbal medicines from home. We use them too. We use them for coughing, stomach ailments and stomach rumbling. They cause vomiting and diarrhea and after that the stomach feels better. We also use if one wakes up and the neck is hurting.

Interviewer: Which health facility do you frequent the most?

Respondent: We go to the Sagamaganga dispensary. If one still feels ill, then we go to Kibaoni health center.

Interviewer: Do you ever go to the ("waganga") traditional healers?

Respondent: Yes, people do (laughter) but those who are witches.
