# Supplementary material for: Lay attitudes and misconceptions and their implications for the control of brucellosis in an agro-pastoral community in Kilombero district, Tanzania
Source: PLoS Negl Trop Dis. 2021 Jun 10;15(6):e0009500. doi: 10.1371/journal.pntd.0009500 (PMC8219154; doi:10.1371/journal.pntd.0009500)
Supplement: Attachment — Submitted filename: PLOS NTDs paper Comments FINAL.docx [file pntd.0009500.s006.docx]

Institute of Anthropology, Gender and African Studies

University of Nairobi

P.O Box 30197

G.P.O Nairobi

Kenya

Phone (+254) 723 813 427

[mwihakicarol@gmail.com](mailto:mwihakicarol@gmail.com)

**7^th^ Nov 2020**

Dear Editors,

Please find attached our revised manuscript *“Lay attitudes and misconceptions and their implications for the control of brucellosis in an agro-pastoral community in Kilombero district, Tanzania”*. Below is a point by point response to all the reviewers’ comments.

Kind regards,

Caroline Mwihaki Mburu

**Overview of the main revisions made:**

We thank all the reviewers for taking their time to review this manuscript. We are grateful for all their comments, inputs and suggestions. We agree with the reviewers’ main concerns and therefore we synthesized the presentation of the quantitative and qualitative data for more cohesiveness. We also changed our methods of quantitative analysis to better present the data. Another main area of revision was clarifying the study design and methodology. The abstract and the author summary have been revised too.

All the page and line numbers here correspond to the line numbers in the revised manuscript. All the changes to the manuscript have been indicated by using track changes.

**Point by point answers to the reviewers**

**1.** Reviewer #1: Generally, lme4 is used for generalized linear mixed effect models setting random effects. However, the analysis was performed by glm. Explain why lme4 was used.

*Thank you for this observation, reporting the use of the lme4 package for R was incorrect. We revisited the quantitative analysis and re-analysed the data for knowledge on brucellosis and risk practices. For the analysis of knowledge score as response variable we used a zero-inflated regression model with negative binomial distribution (ZINB) to test for the relationship between knowledge and level of knowledge on brucellosis and socio-demographic factors. Since 93% (309) of participants answered that they had never heard of brucellosis and therefore scored zero in all knowledge questions, we identified the data as over dispersed due to the presence of excess zeroes. The data analysis section has been modified accordingly (page 6, line 26- page 7 line 14). Results were added in tables 4 and 5 on pages 13 and 14 respectively. Practice scores were transformed into proportions and a generalised linear model with a quasibinomial distribution was used. Response variable was the proportion of risky practises undertaken of the total number of risky practices mentioned in the questionnaire.*

**2.** I think binomial distribution should be used instead of Poisson distribution.

*Please see the answer to question #1 above.*

**3.** P10 line 17
“over 50 years of age” This result just shows the analysis of comparison between age category 18-25 and all other age categories. If the authors’ interest is to compare the youngest category and other categories, keep current form of analysis but revise the sentence, taking my comment into account. If the authors hypothesis is that knowledge or practice improve as people get older, change the age categories into rank (for example 18-25:1, 26-33:2, 34-41:3, …) and analyze it by glm. The same goes for education.

*Thank you for this suggestion. We changed the age and education categories into rank and report the results of the ZINB and glm in tables 4 and 5.*

**4.** Reviewer #2: This paper addresses an important area of focus, namely, the interface between livestock and humans, especially for pastoral/agro-pastoral communities especially across Africa. In Kenya and Tanzania this is especially important for the Maasai due to there close association with livestock. In addition, the Maasai also inhabit areas which bring them into considerable interaction with wildlife. The study explores the disparities in risk perception and the practices that raise the risk of transmission in an agro-pastoral setting. In my view, this objective is clearly stated.

*Thank you.*

**5.** The study design is appropriate and the study population clearly described.

*Thank you.*

**6.** It is not clear what the sample size is. On page 5 line 20 the authors indicate that 333 structured interviews and 7 focus group discussions were done. However, on the same page 5 line 25, the authors indicate that all 352 agro-pastoralist households were visited. This requires clarification.

*Thank you for the crucial observation. This has been clarified on page 5 lines 18-24. A total of 176 agro-pastoralists households were visited in an aim to interview one male and one female in each household which would have led to a total of 352 study respondents. However, 333 respondents were interviewed from 167 households, the rest being unavailable.*

**7.** Some of the aspects/data collected for this work, e.g. wildlife-livestock-human interface (page 6 line 9) has not come out in the data presented and, hence, in the discussion.

*In table 6 under question 11 “do you graze your livestock in areas with wildlife?” 42% of the respondents said yes. This data is also presented in page 17 lines 9-20 and discussed in page 20 line 31 to page 21 line 5.*

**8.** The authors indicate (page 7 line 21) that "For practices, the respondent reporting the riskiest practices scored the highest." What about the scores for knowledge? Was knowledge also similarly ranked?

*All practices risking transmission were weighted equally and the same applied to knowledge. We changed the wording to “the respondent reporting to perform the highest number of risky practices scored the highest” to clarify this on page 7 line 22-23. Answering “yes” to performing a practice associated with risk of transmission contributed 1 point toward the overall score. The same was done for the knowledge score.*

**9.** Reviewer #3: - The objectives are clear
- The design is well articulated 
- The population is appropriate
- Sample size is okay
- The analyses need revistation

*Thank you.*


**10.** Reviewer #1: Demographic characteristics
Show all the data by table or figure. The information about age and ethnic group are unclear. In terms of ethnic group, quite small group, less than 5 people for example, can be grouped as other group, but otherwise show all the data.

*This information has now been added in Table 1 on page 8.*

**11.** P8 line 12-13
Traditional religion (56%) is inconsistent with the data in EXCEL file. Carefully check this contradiction.

*During the qualitative phase of this study, after the structured interviews were conducted, we observed that atheism was not practiced in the strict use of the term rather they had a traditional religion which is what is presented in the results. On the other hand, this interview was conducted in Kiswahili and included the term for “not believing in any God as identified by the major religions” which most of the respondents identified with because they had a local traditional religion which did not fit in any of the category of major world religions.*

Most of them identified-----” Do not use “Most” for 56%.

*We changed this on page 8 line 13 .*

**12.** P8 line 19-20 “their causes were not associated with brucellosis as shown in Table 2”
Current expression means causality between brucellosis symptoms and its cause was cleary identified by robust, scientific methods. But this is based on questionnaire survey so the true cause is not identified. Rephrase it.

*We rephrased this on page 10 lines 9 to 10..*

Does Table 2 mean Fig 2?

*Thank you for this observation; this has been adjusted to table 3 on page 10.*

**13.**Table 1
The readers have an impression that tables of awareness and causes have some relations since they are expressed in parallel. Rearrange table.

*Thank you, for clarity this table has now been revised to include 2 separate tables; table 2 on page 9 and table 3 on page 10.*

**14.** In the table for awareness, the relationship between question and frequency is a bit unclear. Revise it.

*We followed your advice and revised this in table 2 on page 9. This table shows the percentage of respondents who responded to each of the questions assessing their awareness on brucellosis.*

**15.** P10 line 2-3
It’s not clear for me to show the data from just the 24 respondents who had heard of brucellosis. If the authors want to show the major brucellosis symptoms recognized by farmers, the data from all farmers should be used.

*In this study, only 24 respondents had heard of brucellosis and thus could talk about any symptoms they assign to the disease. Our aim was to see what symptoms the farmers associated with brucellosis. The rest of the respondents could not talk about the major brucellosis symptoms because they had never heard of brucellosis and were unaware of the disease or its symptoms.*

**16.** Fig 2
In title, it’s unclear what “minority” means. Revise the title.

*We clarified this in the revised Fig 2 on page 10.*

“Percentage” should come to left side.

*We labelled the y-axis accordingly*.

**17.** P10 line 33-P11 lin2
The method of analysis is not described.

*We changed the method of analysis and the presentation of the results in page 13 lines 13-15.*

**18.** Table 2
Show the category to compare in Explanatory variables. Except for sex, the category compared is not clear.

*The reference category for sex has now been indicated in tables 4 and 5 on page 13 line 17-18 and page 14 line 1-2 and variables education and age have been transformed into rank and therefore do not have a reference category.*

**19.** In CI, it’s not clear whether “-” shows minus or range of CI. It seems there are mistakes. Revise it.

*We have replaced the tables in the results section together with the analysis and hope it is now clear that the CI is a range.*

**20.** Practice-Education (secondary): Coefficient or CI is wrongly expressed.

*We have revised the analysis section and the corresponding results section.*

**21.** Table 3 is not found.

*Apologies for this anomaly. The tables have all been revised and numbered correctly on pages 8,9,10,13 and 14.*

**22.** Table 4
I do not think this is from the final model since all the explanatory variables are included.

*This table has been changed with the revised analysis. All the variables contributing to the model fit have been included.*

**23.** P16 line10
What are the sources of the data?

*This data is from the structured interviews which we have now indicated on page 17, line 9.*

**24.** P17 line31 
“since they owned large herds” The logic is unclear. Clearly explain it.

*Thank you for this observation. In this context the number of animals kept was more important than the quality of animals in the herd because of the status this provided to the farmer. This means that less than optimal animals were retained because of other perceived cultural benefits. This has now been explained on page 18 lin33 to page 19 line 4.*

**25.** P18 line26
“Positive” is unsuitable word here. Rephrase the sentence.

*Rephrased. Page 19 line 30 to line 31. .*

**26.** P18 line27-29
The logic is unclear. Please clarify it.

*Rephrased for clarity. Page 19 lines 31 to page 20 line 1.*

**27.** P19 line15-20
Describe the information about age in the references.

*The information about age is according to the findings of this study which is corroborated by other studies as explained on page 20 lines 22 to 26.*

**28.** Reviewer #2: The authors present the results starting first with quantitative and followed with qualitative data. The captions for the Tables and Figures should be more succinct. It is also important to include the N in the Tables in order to put the percentages into perspective.

*The presentation of the results has been changed so that quantitative and qualitative results are presented together in a more coherent manner. The captions for the tables and figures have been made more concise. N has been included in all the tables too.*

**29.** Table 1 (page 8) is difficult to follow. For instance, for have you heard of brucellosis in livestock the frequency is 7.2% (it would be nice to indicate the actual count as well). Now, for the perceived causes, which is given next, do those percentages derive from the 7.2%(n??) respondents? One is unable to make this decision from the Table.

*We revised the analysis and two tables 2 and 3 on pages 9 and 10 respectively were generated that add clarity.*  **30.** The caption for Table 2 is a too long (it is, in fact, two sentences long). The table also includes under practice Ethnicgroup1. What is the justification of including this as a practice?

*The title has been made concise as suggested. The rationale for including ethnicity is to reflect our investigation of whether the performance of riskier practices is based on the participants ethnicity.*

**31.** Where is Table 3 in the paper?

*We apologize for this oversight. All the tables in the paper have been adjusted.*

**32.** The caption for Table 4 also should also be made more succinct.

*Done.*

Also for this Table, I am looking for an explanation of the link between education and religion in terms of their relationship with practices. Could we get some additional information on this, especially for the qualitative data?

*The study found that the performance of fewer practices with transmission risk was associated with religion and level of education. Based on the qualitative data, including the FGDs, culture was a bigger indicator of behavior than religion and education, because of low risk perception of zoonotic diseases and because these practices were a way of life and deeply entrenched.*

**33.** I fail to get the rich narrative that should come from the qualitative data. At best there are many short quotations but the authors seem to have failed in getting the story out in a clear and concise way supported by the quotations. Reading through the qualitative section, one is not sure whether their was a more general concurrence from the 7 FGDs and, which is represented by the quote or not. The authors should review this section.

*We reviewed this section following your suggestion and integrated the qualitative and quantitative data. More depth has been added to the qualitative data too.*

**34.** On page 16 line 12: I am still trying to figure out how cattle interact with ... hippos and lions ... How is the term interaction used in this sense?

*Done. This has been changed to “routinely encounter wild animals” on page 17, line 11.*

**35.** The caption for Figure 2 should be reviewed and include the percentages to provide a little more information.

*We revised fig 2 and added a clearer title on page 10 line 1.*

**36.** Reviewer #3: - The qualitative part will need improving

*The results section has been reworked so that the quantitative and qualitative data are presented alongside each other in a more coherent fashion.*

**37.** Reviewer #1: Describe the limitations of the study.

*We added a paragraph on the limitations on page 22 lines 9 to 17.*

**38.** Reviewer #2: What are the limitations, if any, for this study?

*We added a paragraph on the limitations on page 22 lines 9 to 17.*

This study has significant implications on communication of public health messages. The authors should build a case based on the data presented. At present one cannot get the public health argument. On page 20 line 20 it is indicated that "This disparity in risk perception, thus, presents a challenge in risk communication related to animal handling and consumption of animal sourced foods." What disparity is being referred to here? In the previous sentence the authors write about the disparity in risk perception between the health experts and lay people. One may argue that the disparity is always there between the experts and lay people. How this creates a challenge in risk communication is not clear. In my view, a challenge in risk communication would arise if the lay people have two or more competing risk perceptions. This should be clarified.

*Thank you for your suggestions on clarification. We addressed this on page 21 line31 to page 22 line 4. The findings of this study demonstrate that as long as lay people do not consider there to be a problem in their consumption of raw animal products and livestock handling then there is the possibility of miscommunication between professionals and the community. Consequently, it is important to not only sensitize the community but engage them to address their concerns and misconceptions.*

**39.** Reviewer #3: - The conclusions are okay however there are many limitations and have not been well addressed

*Thank you. The limitations of this study have now been included on page 22 lines 9 to 17.*

**40.** Reviewer #1: P1 line 21-22
The number of FDGs is complicated.

*We clarified this on page 1 lines 18-20.*

**41.** P2 line 33
“questions and concerns” This should be described in detail in discussion.

*Done on page 22 lines 1 to 4.*

**42.** Introduction
In the current form the readers have an impression that brucellosis is endemic just in sub-Saharan Africa but the disease is also endemic in other areas. Rephrase Introduction.

*We clarified this on page 2 lines 29-31.*

**43.** P3 line30
The reference 13 does not describe the information.

*We apologize for that error. Done on page 3 line 19.*

**44.** P3 line 34-P4 line4
Epidemiological study and implementation of disease control are largely different. Describe the examples of the implementation of disease control and the results.

*This has been done on page 3 lines 24-32.*

**45.** The information is complicated among Study design, Sampling and Data collection. For example, if this is a census among the 3 villages, explain it before the number of interviews. Avoid repeating same information and reorganize these parts.

*We have reviewed this section for clarity on pages 5 line 11 to page 6 line 14.*

**46.** Describe the reason not to include some agro-pastoral households in structured interview.

*The reason some were not included is simply that they were unavailable during the data collection period. This has now been clarified on page 5 lines 20 to 21.*

**47.** Men and women were interviewed separately in FGDs. Make it clear whether the authors count this separated discussion as one or two.

*A total of 4 male and 3 female groups were engaged in separate FGDs, yielding a total of 7 FGDs as described on page 5 lines 30 to 34.*

**48.** Describe how many participants joined in a FDG.

*Each FGD had between 8-12 participants as indicated on page 6 line 1.*

**49.** P6 line34 - p7 line3 
Make it clear the explanatory variable and response variable.

*We clarified the variables. Page 7 lines 7 to 10.*

**50.** Show all the 29 and 12 categories for knowledge and practice levels, respectively. Either in methods or results is fine.

*The knowledge and practice levels correspond to the number of questions answered. We clarified this on page 7 lines 16 to line 23.*

**51.** Cleary describe univariable and multivariable analyses in method and result. It is not clear which result is shown in the table in result part.

*We revised the analysis of the quantitative data and added the information to methods and results.*

**52.** P7 line21-22
The sentence is not clear for me. Rephrase it.

*Done on page 7 lines 22 to 23.*

**53.** P7 line 33 
Attach the reference for “supported by existing literature”

*Done. Page 8 line 1.*

**54.** Reviewer #2: The paper require editing through out. In particular, the Table and Figure captions should be reviewed. The titles could be made more succinct. I have made additional suggestions in the preceding sections.

*The paper has been edited throughout including the presentation of the study findings. The table and figure captions have been made succinct.*  **55.** Reviewer #3: (No Response)

*Well noted.*

**56.** Reviewer #1: Although the data collected in the study is important, the current manuscript has weaknesses in statistical analysis, expression of the results and language quality.
I recommend to have advice for statistical analysis.
I also advise the authors work with a writing coach or copyeditor to improve the flow and readability of the text.

*Thank you for the comment. The paper has been edited to improve the flow. The qualitative and quantitative results have been integrated. Statistical analysis was reviewed.*

**57.** Reviewer #2: This paper provides useful information within a one health approach. The authors have attempted to make an argument on the interaction between wildlife-livestock-human interface but the argument seems lost in the weak link between quantitative and qualitative data. Authors could improve the paper by enhancing the linkage between quantitative and qualitative data.

*Your comment is well noted. The linkage between the quantitative and qualitative data has been improved by presenting the results of both methods of enquiry alongside each other in every section to enhance the flow and cohesion.*

**58.** Reviewer #3: The study aimed to assess perceptions understanding of brucellosis in an agro-pastoral community in Kilombero district, Tanzania. This is a critical study given the insufficient number of social and cultural data on zoonotic diseases. However, one main finding, a total lack of knowledge about brucellosis among the participants, baffles me. Perhaps this emanates from how the data collection was implemented, questions asked, in particular, the qualitative data, as I explain below.

Methodology

a) Having worked with Sukuma and Mangati pastoral groups bordering the Morogoro region, I find it rather odd to read that the interviews were conducted in Kiswahili rather than their mother tongue and without the use of an interpreter (Maasai, possibly, however, the Mangati?). I say these because most are not fluent in Kiswahili (Kindly use the native name Kiswahili), let alone conversing about livestock diseases using a “second” language i.e. Kiswahili. The authors should clearly state this limitation and explain how they think; a clear and meaningful understanding of lay perceptions on zoonotic diseases may have been lost in translation.

*The interviews were conducted in Kiswahili. However, the research assistants were fluent in Kisukuma from which majority of the respondents were drawn and they were on hand to translate. There were very few respondents and participants from the Wamang’ati group. The respondents were very familiar with livestock diseases in general but had very limited information on zoonotic diseases. They actually reported that they had witnessed signs associated with brucellosis but they did not know about the disease and so they attributed these signs to other causes mainly trypanosomiasis, a disease transmitted by tsetse flies causing some similar symptoms.*

b) In addition, it is not stated whether the FGDs were grouped according to the ethnic groups of the participants and not. Why was this not done? And about my observation above, if they were grouped together, how possible was the moderation given the diversity of the participants' indigenous language?

*The FGDs were not grouped according to ethnic groups but by villages. First, the number of agro-pastoralists from the other ethnic groups other than the Wasukuma was small. Secondly, as this was ethnography, the researchers were able to obtain ethnic specific information through participant observation and in depth interviews. Majority of the FGD participants were able to express themselves fluently in Kiswahili and the research assistants translated especially for the older people who preferred communicating in their mother tongue.We added information to the methods section to make the study design clearer.*

c) It would be interesting to know how exactly, even in Kiswahili, the idea of brucellosis was communicated to the participants during the discussions. If the guide only queried about zoonosis as a category of diseases that humans get from livestock, then a lot of lay information may have been missed. In the transcriptions attached, the interviewer asks the respondents if they have heard about brucellosis. What Kiswahili word was used? Does it even exist? Wouldn’t the answer obviously be negative? If participants knew brucellosis in animals, should we read that the same understanding “abortions”, was communicated to the participants and the results could be wrong? No wonder the results section on brucellosis perceptions is so short (line 28-34) but it carries the study.

*The idea of brucellosis was communicated as “ugonjwa wa kutupa mimba”, the local expression for the disease, as well as “homa ya maziwa” and “brusela” which are all commonly used to define brucellosis in Tanzania and are used on awareness posters. The majority of them knew and had witnessed abortions in their livestock but they did not associate this with brucellosis rather with trypanosomiasis and other factors. Before the structured interviews were conducted, key informant interviews with local veterinary and public health officials had shown that brucellosis was not well known among the livestock keepers. The majority of them were familiar with diseases such as foot and mouth and trypanosomiasis and very few had heard of the disease brucellosis.*

Percentages have been used in the qualitative results section in a rather odd fashion, yet this was not clearly justified or referenced in the qualitative data analysis strategy section. I think they do not add any value given that there is a whole section on quantitative results already. If these are from the KAP survey results and were used to elaborate on the quali data, then the design would have to be re-written to conform to the principles of the Mixed methods approaches. A number of frameworks do exist to guide on the same (For example Creswell, J. W., & Clark, V. L. P. (2017). Designing and conducting mixed methods research (Third). Thousand Oaks, CA: Sage publications.)

*Thank you for the comment. The whole section on the study findings has been revised for clarity and better flow.*


The qualitative results could be improved through thoroughly unpacking comparatively notions of zoonotic diseases in the pastoral groups involved, given that the study clearly demarcated them in terms of their ethnic groups (Maasai, Wamangati and Sukuma). It would be interesting to the readers to know how each ethnic group discussed brucellosis instead of lumping lack of understanding across all the ethnic groups. I say this because brucellosis tests are now widely available in many district hospitals, and as one of the study quoted found that the knowledge of some zoonotic diseases among the Maasai, for instance, is increasing because of these health facilities visitations and upon receiving diagnostic results. For example, as a result, some pastoralists and agro pastoralists in some areas of Tanzania now widely speak of “brusela” (localized word) as a “new” disease, emanating from the consumption of livestock products such as milk. To improve the paper, perhaps it will be useful to obtain information from the surrounding health facilities whether such tests are available or have been performed.

*Thank you for your valuable insights. The qualitative results have been improved to provide more detailed information on the lay perceptions related to brucellosis. This includes providing more specific information especially on the two major ethnic groups interviewed the Wasukuma and Wamaasai. In addition, through key informant interviews with public health officials at all the relevant levels, we found that testing for brucellosis was available at the hospitals but the officials noted that this testing is not routinely done because even the practitioners themselves did not often suspect brucellosis in many patients presenting with a febrile illness. The key informants both from the public health and veterinary sectors also noted that brucellosis was not as prioritized even in community sensitization as malaria and HIV/AIDs in humans and foot and mouth disease and Contagious Bovine Pleuropneumonia in Cattle for example. This could have contributed to the low awareness about this disease.*
