# Supplementary material for: Lay attitudes and misconceptions and their implications for the control of brucellosis in an agro-pastoral community in Kilombero district, Tanzania
Source: PLoS Negl Trop Dis. 2021 Jun 10;15(6):e0009500. doi: 10.1371/journal.pntd.0009500 (PMC8219154; doi:10.1371/journal.pntd.0009500)
Supplement: Attachment — Submitted filename: PLOS NTDs Comments Final Feb 2021.docx [file pntd.0009500.s007.docx]

Institute of Anthropology, Gender and African Studies

University of Nairobi

P.O Box 30197

G.P.O Nairobi

Kenya

Phone (+254) 723 813 427

[mwihakicarol@gmail.com](mailto:mwihakicarol@gmail.com)

**23rd Feb 2021**

Dear Editors,

Please find attached our revised manuscript *“Lay attitudes and misconceptions and their implications for the control of brucellosis in an agro-pastoral community in Kilombero district, Tanzania”*. Below is a point-by-point response to all the reviewers’ comments.

Kind regards,

Caroline Mwihaki Mburu

**Overview of the main revisions made:**

We thank all the reviewers for taking their time to review this manuscript. We are grateful for all their comments, inputs and suggestions. We agree with the reviewers’ main concerns and therefore we have revised and edited the quantitative data analysis and the subsequent sections.

All the page and line numbers here correspond to the line numbers in the revised manuscript. All the changes to the manuscript have been highlighted for ease of reference.

**Point by point answers to the reviewers**

**Reviewer 1:**

1.P6 line 7

Move to the results part

*Thank you for this observation. This was deleted as it was already in the results section page 8 lines number 9-11..*

2.Less than 5 for a people group should be grouped as other. The Wamaasai should be separately listed in the demographic characteristics in Table 1.

*We revised the manuscript categorising the Wamaasai as a distinct category in the demographics table and considered them as a distinct group in the analysis. The results of the analyses did not change for knowledge. However, belonging to the Wamaasai significantly increased using risky practices.*

**Data management and analysis**

3.Explain the statistical analysis in more detail and avoid redundancy throughout this part.

*We changed the wording in the data management and analysis section to avoid repetition and to clarify the approach as shown in pages 6 to 7.*

4.Education and age group have more than 2 categories. Explain which data type was used for these explanatory variables in the analysis eg nominal scale or ordinal scale.

*Both education and age-group were used as ordinal variables. A sentence has been added to the methods section to reflect this on page 6 lines 29-30..*

5.Show the 29 and 12 categorical variables for knowledge and practice score and the simple results of them before the statistical analysis.

*Thank you for this suggestion, we have added Table 2 in the paper showing the quantitative variables for knowledge and practices.*

**Results**

6.Table 2

The answers of categories 2-5 are affected by the existence and the frequency of the symptoms in the cattle herd. It is not suitable to show the knowledge of the disease name and observation of the symptoms in a same table with the current title.

*We have re-done this table; now Table 3 with questions on awareness* *separated from questions on the experience of symptoms.*

7.Fig 2

It is not clear what the figure shows due to inaccurate title. Moreover, integrate how to express the result with other data if the figure is necessary.

*Thank you for this observation. We decided to include the signs of brucellosis noted by the 24 respondents who had ever heard of brucellosis in Table 2 with other quantitative variables for clarity and ease of understanding.*

8.P10 line 10

They were not linked to brucellosis. Revise this sentence.

*Done. We revised this sentence as suggested in page 11 line 12.*

9.Table 3

It seems the respondents were asked to choose one category of the cause of each symptom because the total % is 100 in each symptom. Questionnaire interview was incorrect because it excluded the possibility that the respondents knew several causes of the symptom.

*The data for table 3 is sourced from an open-ended question, where most respondents either said they did not know the cause for a symptom or that the symptoms were due to trypanosomiasis. The qualitative data does corroborate the findings that brucellosis symptoms, though witnessed, were associated with other causes, mainly trypanosomiasis. We do not think the information from this survey should be dismissed. The purpose of our mixed study was for qualitative and quantitative data to clarify the issue and give a fuller picture. The data also revealed that most of the livestock keepers interviewed really did not consider these reproductive health challenges in livestock as problematic, as they were regarded as a normal part of life just like infertility and miscarriage in women. The option to choose causes from a list would have resulted in distorted data.*

10.Table 4

Relative risk is only used for cohort study or randomized control trial. Explain why relative risk is used.

*This was a labelling mistake. We changed relative risk to odds ratio to describe any factors increasing or decreasing the odds to be in the group of having no knowledge at all. The estimates, p-values and Cis do not change.*

11.Tables 4 and 5

Show all the results including non-significant factors.

Clearly explain whether the result is from univariate or multivariate analysis.

*We specified that the results are from multivariate analysis in the data management and analysis section.*

12.Refer other papers to learn how to express the results of statistical analysis.

*We consulted other publications to see how they have expressed their results.*

**Editorial and Data presentation modifications**

13.P1 Line 27-28

Here is inconsistent with the results.

*For clarity we started with the symptoms experienced in the herds followed by the livestock keeper’s awareness on brucellosis.*

14.Introduction P3 Line 19

The information about ref 15 is not enough.

*Thank you, we updated the wording of the sentence and the reference has been updated on page 3 lines 18-19.*

**Summary and General comments**

15.The manuscript still has room for improvement in statistical analysis, expression of the results and language quality.

*Thank you for your guidance, we revisited the analysis, results presentation and the language.*

**Reviewer 2**

16.The authors have made a good attempt to respond to the issues I raised in the first review.

Authorship. What role did Dr Khamati Shilabukha play as author?

*Thank you. Apologies for the oversight. Dr Khamati’s role in the study has been included in page 25 under author contributions.*
