# Supplementary material for: Lay attitudes and misconceptions and their implications for the control of brucellosis in an agro-pastoral community in Kilombero district, Tanzania
Source: PLoS Negl Trop Dis. 2021 Jun 10;15(6):e0009500. doi: 10.1371/journal.pntd.0009500 (PMC8219154; doi:10.1371/journal.pntd.0009500)
Supplement: Attachment — Submitted filename: PLOS NTDs Comments May 2021.docx [file pntd.0009500.s008.docx]

Institute of Anthropology, Gender and African Studies

University of Nairobi

P.O Box 30197

G.P.O Nairobi

Kenya

Phone (+254) 723 813 427

[mwihakicarol@gmail.com](mailto:mwihakicarol@gmail.com)

**13^th^ May 2021**

Dear Editors,

Please find attached our revised manuscript *“Lay attitudes and misconceptions and their implications for the control of brucellosis in an agro-pastoral community in Kilombero district, Tanzania”*. Below is a point-by-point response to all the reviewers’ comments.

Kind regards,

Caroline Mwihaki Mburu

**Overview of the main revisions made:**

We thank all the reviewers for taking their time to review this manuscript. We are grateful for all their comments, inputs and suggestions. We agree with the reviewers’ main concerns and have revised and edited the quantitative data analysis and incorporated the other suggestions.

All the page and line numbers here correspond to the line numbers in the revised manuscript. All the changes to the manuscript have been highlighted for ease of reference.

**Point by point answers to the reviewers**

1.Reviewer #1: Page1 line 22
“gender” is vague. Revise the sentence to clarify which sex has positive or negative association.

*Done in Page 1, line 22.*

2.Page1 line24-25 
“Following traditional religion and belonging to the Wamaasai ethnicity increased the odds of carrying out these practices.”
It is not clear whether the result was significant. If not, delete it.

*Done in Page 1, line 25-26.*

3.Page1 line28-30 
“7.2%” is only for the 1st item, not for 2nd and 3rd items within the sentence. Revise it.

*Done in Page 1, lines 29-32.*

4.P6 line24-26
It is not clear which model was used for what association. Cleary explain it. Moreover, if ZINB model was used for the association between socio-demographic variables and practices, it is wrong.

*Thank you for pointing this out. We have clarified which model was used for what association (P7, lines 4-6). We used a glm to check for associations between demographic variables and practices, not a ZINB. The text has been changed to: “Associations between socio-demographic variables and the level of knowledge of the disease were investigated with a zero-inflated negative binomial (ZINB,) model, while involvement in practices risking transmission was modelled using a generalized linear model.”*
5.P6 line29-30
Describe how education and age were changed into ordinal scale.

*Age was transformed into age groups as ordinal numbers with ages 18-25 as group 1, 26-33 as group 2, 34-41 as group 3, 42-49 as group 4 and ≥50 as group 5. Education was changed into discreet ordinal numbers with 0 standing for no education, 1 for primary school education, 2 for secondary education and 3 for college/university education.* *We clarified this on p7 lines 8-14.*

6. P7 line14-25
This should be expressed before the explanation of analysis and models. Data management and analysis part is not organized well. Put the information in order.

*Done in the data management and analysis section.*

7.P7 line16
Remove “s”. This does not appear anywhere else.

*Done in Page 6, line 29.*

8.P7 line22-25
Delete. The sentence should be in Result and there is.

*Done.*

9.Reviewer #2: None. My earlier comments had been addressed in the previous revision.

*Thank you. Noted.*

10.Reviewer #1: Tables 1-4
Integrate the format throughout the tables.

*Done for all tables.*

11.P9 line5-7
This should be in Discussion, not in Result.

*This has been changed to reflect the results. The interpretation has been removed.*

12.P9 line7, Table 2

*This was changed to “Not conceiving/siring” to clarify.*

13.P11 line2-3, line11-12
Here quantitative results should be mentioned. No need to describe qualitative comment.

*The wording has been changed.*

14.Table 3 
It is not clear which answer was returned. The result of second category is incorrect.

*All the participants were asked whether they had experienced any brucellosis symptoms in their herds in the past one year regardless of if they had ever heard of brucellosis or not.*

15.Table 2
This is not well organized. Current table seems to be original format of the questionnaire which is not suitable for readers. Organize it to be easily understood.

*Done. Table 2 revised.*

16.Table 4
This is my previous comments to the Table.
“It seems the respondents were asked to choose one category of the cause of each symptom because total % is 100 in each symptom. Questionnaire interview was incorrect because it excluded the possibility that the respondents knew several causes of the symptom. “
The authors’ answer does not make sense, and the association with the result of the table and qualitative data is not the issue here. If the authors want to use the result, limitation of the interview and thus the result should be clearly mentioned. The readers do not think the result is from open question, and it should have been even possible for the interviewees to answer several causes of symptoms in open question, which was not considered in the survey.
Judging from the authors’ comment, not only this result but all results are now doubtful. Precise explanation about the questionnaire items and the structure of the questionnaire (open/closed question for all the items) must be added in Material and methods, and the way of the expression of all quantitative results should be checked again through the manuscript.

*The perceived sources of common signs of brucellosis were posed as open questions. The participants did not tick any boxes. After the data was looked at nobody mentioned more than one source, thus the data was categorized as seen in Table 4. More information on the structure of the questionnaire indicating the open and closed items has been added to Materials and Methods.*

17.Tables 5 and 6
Since the table shows the result of multivariable analysis, show the result of final model which includes only significant factors. In that case, the result of two models should be expressed separately because they must have different factors. Be careful that the Odds ratio, 95% CI and p-value will be different between the current table and final model. Since this research did not perform univariable analysis, the factors included in the analysis must be clearly described in Material and methods.

*We have now changed both tables so that the null model and the final model are displayed separately. We added information to materials and methods to clearly state the response and explanatory variables used in the analysis (p7 lines 4-16)*

18.Reviewer #2: my earlier comments had been addressed.

*Thank you. Noted.*

19.Reviewer #2: my earlier comments had been addressed.

*Thank you. Noted.*

20.Reviewer #2: In this revised version, the authors have responded to my concern.  I am now satisfied.

*Noted.*
